# Catalase impairs *Leishmania mexicana* development and virulence

**DOI:** 10.1080/21505594.2021.1896830

**Published:** 2021-03-16

**Authors:** Jovana Sádlová, Lucie Podešvová, Tomáš Bečvář, Claretta Bianchi, Evgeny S. Gerasimov, Andreu Saura, Kristýna Glanzová, Tereza Leštinová, Nadezhda S. Matveeva, Ľubomíra Chmelová, Denisa Mlacovská, Tatiana Spitzová, Barbora Vojtková, Petr Volf, Vyacheslav Yurchenko, Natalya Kraeva

**Affiliations:** a Department of Parasitology, Faculty of Science, Charles University, Prague, Czech Republic; b Life Science Research Centre, Faculty of Science, University of Ostrava, Ostrava, Czech Republic; c Faculty of Biology, M. V. Lomonosov Moscow State University, Moscow, Russia; d Martsinovsky Institute of Medical Parasitology, Tropical and Vector Borne Diseases, Sechenov University, Moscow, Russia

**Keywords:** *Leishmania*, virulence, catalase, dixeny, evolution

## Abstract

Catalase is one of the most abundant enzymes on Earth. It decomposes hydrogen peroxide, thus protecting cells from dangerous reactive oxygen species. The catalase-encoding gene is conspicuously absent from the genome of most representatives of the family Trypanosomatidae. Here, we expressed this protein from the *Leishmania mexicana Β-TUBULIN* locus using a novel bicistronic expression system, which relies on the 2A peptide of *Teschovirus A*. We demonstrated that catalase-expressing parasites are severely compromised in their ability to develop in insects, to be transmitted and to infect mice, and to cause clinical manifestation in their mammalian host. Taken together, our data support the hypothesis that the presence of catalase is not compatible with the dixenous life cycle of *Leishmania*, resulting in loss of this gene from the genome during the evolution of these parasites.

## Introduction

*Leishmania* (Kinetoplastea: Trypanosomatidae [1,2]) is a genus of about two dozen species that are responsible for leishmaniasis, a vector-borne disease affecting humans and animals in tropical and subtropical regions [3]. The infection is predominantly transmitted by phlebotomine sandflies and manifests in three different forms – cutaneous, mucocutaneous, and visceral leishmaniasis – depending on the infecting species, geographical location, and the immune status of the host. Currently, the World Health Organization estimates the annual burden of leishmaniasis at about 700,000 to 1 million new cases and up to 65,000 deaths [4]. Although leishmaniasis is a poverty-related disease, climate change, migration caused by the military conflicts, and ever-growing international travel have resulted in the spread of the disease into new areas. The currently employed drugs have numerous side effects and no efficient vaccine is available to eradicate leishmaniasis [5,6]. Thus, functional studies of *Leishmania* are important for our understanding of disease etiology, identification of novel virulence factors, and the development of an efficient vaccination and/or treatment strategy.

*Leishmania* are dixenous (= two hosts) parasites with two main developmental stages: extracellular promastigotes in the insect gut and intracellular amastigotes within vertebrate macrophages. Their life cycle in the invertebrate host is confined to the digestive tract, where elongated nectomonad promastigotes escape from the endoperitrophic space and transform into short leptomonad promastigotes. In the midgut, leptomonads differentiate either into haptomonads attached to the stomodeal valve or into mammal-infective metacyclic promastigotes that are transmitted during blood meals of infected sandflies [7,8]. In the blood, metacyclic promastigotes are phagocytized by neutrophils and macrophages and transform into non-motile amastigotes inside phagolysosomes [9]. Upon ingestion by sandflies, *Leishmania* amastigotes differentiate into procyclic promastigotes inside the insect’s midgut endoperitrophic space, and the whole cycle repeats [10–12].

It is generally accepted that dixenous parasites have evolved from their monoxenous (= one host) relatives [13], but how extensive genomic changes during the evolution of Trypanosomatidae species happened to allow the emergence of such a complex lifestyle is still a matter of debate. On the one hand, the infective potential of presumably monoxenous species was supported by their ability to adapt to the warm-blooded environment in coinfections [14–17] or single infections [18], and further corroborated by the thermo-resistance of some trypanosomatid species [19–21]. On the other hand, comparative genomic analyses of monoxenous and dixenous species have revealed large-scale gene gains and losses, highlighting crucial differences between these groups of parasites [22–24]. The gene for the antioxidant enzyme catalase is on this list. This protein is responsible for the decomposition of H_2_O_2_ and, thus helps to protect from dangerous reactive oxygen species (ROS). We have recently performed a broad genomic screening for the presence of catalase in Euglenozoa [25,26]. The catalase-encoding gene was absent in the common ancestor of trypanosomatids and acquired independently, at least twice, by monoxenous Leishmaniinae and members of the *Blastocrithidia*/”*jaculum*” clade [27]. Surprisingly, it was secondarily lost from all *Leishmania* spp. genomes sequenced so far. This agrees with studies pointing to the signaling role of H_2_O_2_ in regulating *Leishmania* differentiation [28,29]. Taken together, these results prompted us to speculate that the loss of catalase could have been a cause or an effect of the dixenous life cycle evolution in *Leishmania*. To investigate this, we expressed catalase from the monoxenous Leishmaniinae *Leptomonas pyrrhocoris* in the dixenous *Leishmania mexicana*. Of note, expression of catalase in *Trypanosoma brucei* and *T. cruzi* is detrimental for the development of these parasites *in vivo* [30,31]. These cases are not directly relevant for *Leishmania*, because, in contrast to the latter, the common ancestor of trypanosomes did not possess a catalase-encoding gene [32].

Numerous molecular tools for regulated gene expression and/or protein (de)stabilization have been developed over the year, allowing researchers to tackle different aspects of *Leishmania* biology [33–36]. However, most of these systems have shown insufficient efficacy in developmental studies. The main reason for this is the unconventional mechanism of gene expression in trypanosomatids, which relies on the formation of poly-cistronic mRNAs processed further *via trans*-splicing [1,37]. Gene expression heavily depends on the regulatory elements in the untranslated regions (UTRs), controlling the transcript processing, stability, and subsequent translation in each of the main developmental stages of *Leishmania* (procyclic promastigotes, metacyclic promastigotes, and amastigotes) [38–41]. The UTRs in most commonly used *Leishmania* vectors drive expression in specific stages of the parasite’s life cycle [34,42], making developmental studies not possible.

In this work, we applied a new strategy to express catalase throughout *Leishmania* development. Our approach relied on the 2A self-cleaving peptide derived from *Teschovirus A* (previously *Porcine teschovirus-1*) [43]. We demonstrated that catalase-expressing *L. mexicana* are defective both *in vitro* and *in vi*vo (in sandflies and mice infection models), further supporting our premise that catalase presence is not compatible with the dixenous life cycle of *Leishmania*.

## Material and methods

### Ethics statement

Animals were maintained and handled in the animal facility of Charles University in Prague in accordance with institutional guidelines and Czech legislation (Act No. 246/1992 and 359/2012 coll. on protection of animals against cruelty in present statutes at large), which complies with all relevant EU guidelines. All the experiments were approved by the Committee on the Ethics of Laboratory Experiments of the Charles University and were performed under permission No. MSMT30397/2019-3 of the Czech Ministry of the Environment. All efforts were made to minimize the number and suffering of experimental animals during the study.

### Leishmania mexicana *axenic cultivation and differentiation*

*L. mexicana* (MNYC/BZ/1962/M379) promastigotes were cultured in M199 medium (Sigma-Aldrich, St. Louis, USA), supplemented with 2 µg/ml biopterin (Sigma-Aldrich), 2 µg/ml Hemin (Jena Bioscience GmbH, Jena, Germany), 25 mM HEPES and 50 units/ml of penicillin/streptomycin (Life Technologies/Thermo Fisher Scientific, Carlsbad, USA), and 10% heat-inactivated fetal bovine serum (FBS, BioSera Europe, Nuaillé, France). Metacyclic promastigotes and amastigotes were differentiated as previously described [44]. Prior to *in vivo* analysis, *Leishmania* cell lines were passaged through mice. Freshly isolated promastigotes were cultured in M199 medium (Sigma-Aldrich) containing 10% heat-inactivated FBS (Life Technologies) supplemented with 1% Basal Medium Eagle vitamins (Sigma-Aldrich), 2% sterile human urine, and 250 μg/ml amikacin (Bristol-Myers Squibb, New York, USA). For mice infections, the pH of the cultivation medium was adjusted to 5.5.

### Sandflies and mice

The colony of *Lutzomyia longipalpis* (originated from Brazil) was maintained in the insectary of the Department of Parasitology, Charles University in Prague, under standard conditions as described previously [45]. BALB/c mice (AnLab, Prague, Czech Republic) were maintained in the animal facility of the Department of Parasitology with a 12/12 light/dark photoperiod, at 22–25°C, and 40–60% humidity.

### *Sequence analysis of* CATALASE

The *CATALASE* sequences (*Homo sapiens* NP_001743, *Leptomonas pyrrhocoris* LpyrH10_15_0020, *L. seymouri* Lsey_0026_0490, *Crithidia fasciculata* CFAC1_250006200, and *C. thermophila* ANJ89234) were retrieved from GenBank of TriTrypDB [46,47]. They were aligned with MAFFT v. 7.471 [48] using G-INS-i iterative refinement method. NCBI-CDD [49] was used to search for the conserved domains and sites.

### Expression vectors and transfection

The genetic nomenclature used in this work followed the guidelines of Clayton et al., 1998 [50]. All constructs described below were sequenced before transfection.

To analyze the expression of the *B-TUBULIN* locus, a construct to replace *LmxM.32.0792* with the hygromycin B (*HYG*) resistance gene ORF was generated by fusion PCR [51,52]. 5´and 3´*Β-TUBULIN* UTRs, and *HYG* ORF (from pLEXSY-hyg2, Jena Bioscience GmbH) were amplified using primer pairs A1/B1, C1/D1, and E1/F1 (hereafter all primer sequences are listed in S1 Table). Nested primers G1/H1 were used for the last PCR amplification step. The PCR product was gel purified and transfected into the log phase-grown procyclic promastigotes of *L. mexicana* as described before [53]. The transfectants were selected in cultivation medium supplemented with 100 μg/ml of hygromycin B (Carl Roth GmbH, Karlsruhe, Germany) for 10 days.

The *Teschovirus A* P2A DNA fragment was generated by conventional PCR with long overlapping oligonucleotides (A2/B2). It was fused with *LmxM.32.0792* 5´and 3´ UTRs (amplified with C2/D2 and E2/F2, respectively) using the nested primers G2/H2. The final product was cloned into the *Swa*I-digested pLEXSY-sat2.1 (pLEXSY_*LmxM.32.0792*_UTR). Tagged nourseothricin resistance gene (*V5::SAT*), *mCHERRY* (*mCHERRY::HA*), and *CATALASE* (*CAT::HA*) ORFs were PCR amplified from the pLEXSY-based constructs using primers A3/B3, C3/D3, and E3/F3, respectively. They were cloned into the pLEXSY_*LmxM.32.0792*_UTR using restriction enzymes *Eco*RI and *Hind*III for *V5::SAT*, and *Bgl*II and *Kpn*I for *CAT::HA* and *mCHERRY::HA*, thus producing cassettes referred to as *SAT::2A::CAT* and *SAT::2A::mCHERRY*. Restriction sites for cloning were introduced into the primer sequences. The resultant plasmids were digested with *Swa*I, DNA products were purified and used for transfection as described above. The transfectants were selected in cultivation medium supplemented with 100 μg/ml of nourseothricin (Jena Bioscience GmbH) for 10 days.

### Verification of genome integration by PacBio sequencing

*L. mexicana* genome (MNYC/BZ/1962/M379) with *SAT::2A::mCHERRY* construct was sequenced using the PacBio RSII technology at Macrogen Inc. (Seoul, Korea), yielding a total of 102,977 polymerase reads with an average polymerase read length of 12 kbp. These data were used to assemble a full reference genome with Canu v. 2.0 [54]. Exonerate v. 2.2.0 [55] was used to annotate (alignment model: protein2genome, percent of query coverage >85%) that locus by homology with *LmxM.32.0792* protein sequence. Furthermore, we picked PacBio reads that had *SAT::2A::mCHEERY* sequence using BLASTN from NCBI-blast-2.3.0+ package [56]. In total, 13 reads were picked and separately reassembled with Canu. This procedure yielded a 6,602 bp-long contig, which represented the allele variant of the *Β-TUBULIN* locus with one *Β-TUBULIN* gene replaced with *SAT::2A::mCHERRY* sequence. As overall coverage of this contig was low, the assembler was not able to correct all sequencing errors in a consensus sequence. Alignment of the 6,602 bp-long contig with *Β-TUBULIN* locus was done manually.

Similarly, we performed PacBio sequencing (but with lower sequencing depth, 13,068 polymerase reads) for *L. mexicana-*CAT. We mapped these reads on the *L. mexicana* genome with BWA tool v. 0.7.12 [57], using “bwa mem” alignment algorithm with “-x” option set to “pacbio”. Average mapped read length was 11 kbp. Only 5% of the genome was not covered and 72% of the genome was covered with less than seven reads. From 16 reads that overlapped with the *Β-TUBULIN* tandem array on chromosome 32 (coordinates 281,233–282,564), only three contained *SAT::2A::CAT* fusion surrounded by proper sequences. These reads were picked and further examined with BLASTN against *L. mexicana* chromosome 32, NCBI non-redundant nucleotide collection, and *SAT::2A::CAT* fusion sequence. BLASTN high scoring pairs were used to annotate PacBio sequences to ensure that they contain a *SAT::2A::CAT* fusion, surrounded by proper *Β-TUBULIN* sequences from the chromosome 32. To ensure that we did not miss any other unmapped PacBio reads bearing *SAT::2A::CAT* fusion sequence (either due to the presence of non-genomic sequence introducing long deletion in alignment or read mapping in other genomic location), fusion sequence was BLASTed against all PacBio reads, but only three reads that had been already annotated, were returned.

### RNA isolation and sequencing

Total RNA was isolated as described previously [23]. Six transcriptomic libraries were prepared using TruSeq stranded mRNA kit (Illumina, San Diego, USA) and were sequenced on Illumina platform in paired-end mode with read length of 150 bp at Macrogen Europe (Amsterdam, Netherland). An average sequencing depth was 31 × 10^6^ reads per sample. RNA sequencing reads were deposed in NCBI SRA under BioProject PRJNA660365.

### Differential expression analysis

RNA sequencing reads for each sample were trimmed using Trimmomatic v. 0.39 [58]. Trimmed reads were mapped on the TriTrypDB *L. mexicana* genome assembly with Bowtie2 v. 2.3.4.1 [59] using default options (end-to-end mapping). Read alignments were processed with SAMTools and BEDTools [60] to extract read counts per genes. Read counts data were analyzed with DESeq2 package v. 1.18 [61]. Read counts were log-normalized by DESeq2 “rlog” function.

### Analysis of target gene expression by RT-qPCR and Western blotting

RNA and proteins from axenically differentiated *L. mexicana* were collected as described previously [62,63]. RT-qPCR analysis was done according to [42], Western blotting was performed using polyclonal anti-HA antibodies (Sigma-Aldrich, 1:1,000) and anti-*Leishmania* serum (1:100), followed by visualization with peroxidase-conjugated anti-rabbit IgG and anti-mouse IgG secondary antibodies (both Sigma-Aldrich) at 1:80,000.

### Catalase activity assays

Catalase activity was determined using a catalase calorimetric activity kit (Life Technologies/Thermo Fisher Scientific) according to the manufacturer’s instructions. The absorbance was read at 560 nm on Epoch Microplate Spectrophotometer (BioTek/Agilent, Winooski, USA). All experiments were performed in triplicate.

For fast evaluation of enzymatic activity, *L. mexicana* cells (5 × 10^6^) were spun down at 3,000 rpm for 5 min, washed with PBS, re-suspended in 10 µl of PBS, and placed on a microscopic slide. A drop of 3% H_2_O_2_ was added to the cells, and the formation of oxygen bubbles indicated catalase activity.

### Cell viability assay

The cytotoxic effect of H_2_O_2_ was determined using the fluorimetric method based on the conversion of resazurin (Alamar Blue) by live cells [64,65]. Briefly, serial 2× dilutions of H_2_O_2_ (4 to 0.03125 mM) were prepared in 96-well plates. The log phase-grown procyclic promastigotes were added to the wells to the final concentration of 5 × 10^5^ cells/ml and the plates were incubated for 1 h. The 0.5 mM stock of resazurin was added to the final concentration of 50 µM. Fluorescence was read after 24 h of incubation at 23⁰C on Infinite M200 (Tecan, Männedorf, Switzerland) using excitation and emission wavelengths of 540 and 590 nm, respectively. The data were analyzed using Prism (GraphPad Software, San Diego, USA) with nonlinear regression and sigmoidal dose-response analysis with variable slope to obtain EC_50_ values. All experiments were performed in triplicate.

### Sandfly infection

Promastigotes from log-phase cultures (day 3–4) were washed twice in saline and re-suspended in heat-inactivated rabbit blood at a concentration of 1 × 10^6^ promastigotes/ml. Sandfly females (5–9 days old) were infected by feeding through a chick-skin membrane (BIOPHARM, Jílové u Prahy, Czech Republic) on the promastigote-containing suspension. Engorged sandflies were separated and maintained under the same conditions as the colony. Females were dissected on day 2, 5, and 9 post-blood meal (PBM) and the abundance and location of *L. mexicana* parasites in the sandfly digestive tract were examined under the light microscope. *Leishmania mexicana* parasites were found in five locations in the sandfly digestive tract: endoperitrophic space (E.SP.), abdominal midgut (AMG), thoracic midgut (TMG), cardia (CA) and the stomodeal valve (SV). Parasite loads were estimated by two methods [66]. Gut infections *in situ* were graded as light (<100 parasites/gut), moderate (100–1,000 parasites/gut) and heavy (>1,000 parasites/gut). For exact counting of parasites, 10 guts from each group were transferred into 100 µl of 1% formaldehyde solution, re-suspended and counted using a hemocytometer. Experimental infections were repeated independently four times.

### Transmission by bite

Anesthetized BALB/c mice were placed individually into small 20 × 20 cm cages and 10–15 infected *L. longipalpis* females (days 8–13 PBM) were allowed to feed on the whole body of mice for one hour. Two researchers followed each experiment; one recorded biting sites and feeding time, while the second collected engorged sandflies by an aspirator immediately after terminating their blood meal. After exposure, mice were euthanized; the biting sites excised and skin biopsies were stored at −20°C until DNA extraction. Engorged sandfly females were dissected immediately and their guts checked for the presence of *Leishmania*.

### Macrophage culture and infection

Bone marrow-derived macrophages were isolated and infected as described previously [67]. Briefly, cells were released with trypsin-EDTA solution (Sigma-Aldrich), washed in 0.9% saline solution and seeded into Greiner Cellstar 24-well plates (Sigma-Aldrich) at a density of 4 × 10^5^ cells/ml. Macrophages were exposed to the stationary-phase grown parasites at a promastigote to macrophage ratio of 6 to 1. Two hours later, complete uptake of *Leishmania* was verified under a light microscope and cells were incubated for 3 days in complete RPMI 1640 (Life Technologies/Thermo Fisher Scientific) supplemented as above, with a combination of IFN-γ (50 U/ml, AbD Serotec/Bio-Rad, Kidlington, UK) and LPS (0.5 µg/ml, Sigma-Aldrich) for classically stimulated macrophages, or IL-4 (25 ng/ml, eBioscience/Thermo Fisher Scientific, San Diego, USA) for alternatively stimulated macrophages. Infected macrophages were then lysed in 0.016% SDS (Sigma-Aldrich) for 5–10 min at room temperature. The suspension was passed through the 1 ml insulin syringe, washed with 0.9% saline solution, and spun down at 3,000 × g at room temperature. The pellet was re-suspended in 100 µl of 0.9% saline solution and amastigotes were counted with a hemocytometer. All experiments were performed independently twice and samples were analyzed in triplicate.

### Nitrite analysis to measure NO production

The accumulation of nitric oxide produced by cultured macrophages over a 72 h period was determined in a microplate assay using Griess reagent as described previously [68].

### Mice infection

*Leishmania mexicana* cultures (passage 1 or 2 after recovering from mice lesion) were enriched for metacyclic forms by inoculation into cultivation medium with the pH adjusted to 5.5 [44] and harvested 10 days later. Eight weeks old female BALB/c mice (10 per WT; 10 per *L. mexicana-*mCHERRY; 20 per *L. mexicana-*CATALASE lines) were anesthetized with the mixture of ketamine and xylazine (62 and 25 mg/kg, respectively) and infected by intradermal inoculation of 1 × 10^5^ parasites in 5 µl of sterile saline into ear pinnae. Control mice were inoculated with the same volume of saline without parasites. Mice were checked weekly for external signs of the disease until week 12 post-infection (p.i.) when they were subjected to xenodiagnostic experiment and sacrificed.

### Xenodiagnosis

Xenodiagnostic experiments were performed on week 12 p.i. Five to seven-day-old *L. longipalpis* females were allowed to feed on the inoculated ear (the site of inoculation of parasites) of anesthetized mice. Mice were covered with the cotton bag, so that only the ear pinnae were accessible to sandflies and placed into a small cage with 30–50 sandfly females. After one hour, engorged females were separated and maintained at 26°C on 50% aqueous sucrose solution. Females were dissected and their guts examined under the light microscope for presence of *L. mexicana* 8 days PBM.

### Morphometry of parasites

Midgut smears of sandflies infected with *L. mexicana* dissected by day 9 PBM were fixed with methanol, stained with Giemsa (Sigma-Aldrich), examined by light microscopy (Olympus DP70, Olympus, Shinjuku, Tokyo, Japan) with an oil immersion objective and photographed. Body length, flagellar length and body width of 500 randomly selected promastigotes from 5 to 6 samples were measured for each group using Image J software [69]. Three morphological forms were distinguished, based on the criteria of [70,71]: (a) nectomonad promastigotes, body length ⩾ 14 µm; (b) metacyclic promastigotes, flagellar length >2 times body length and body length <14 µm, and (c) leptomonad promastigotes, body length <14 µm and flagellar length ⩽ 2 times body length. Haptomonads were not distinguished as they may remain attached to the gut and can be underrepresented on gut smears.

### Statistical analysis

Differences in intensities of infections and percentage of morphological forms found in infected flies were analyzed by the χ2 test using SPSS Statistics 23 package. Differences in parasite loads in sandflies were tested by the non-parametric Mann–Whitney U test. The correlation between lesion size and *Leishmania* strain (categorical variable) over time (continuous independent variable) were tested using R software by fitting multilevel linear regression model (package “nlme”).

## Results

**Novel 2A self-cleaving peptide-based expression system in *L. mexicana*: proof of principle and revised structure of the *Β-TUBULIN* locus**

Our attempts to express a catalase from *L. pyrrhocoris* H10 in three developmental stages of *L. mexicana* using a T7 polymerase-driven, tetracycline-inducible gene expression system failed [42,53]. The main reason for this is that the inducible gene expression system depends on exogenous UTRs flanking both the gene of interest and the T7 polymerase integrated into the *18S rRNA* locus, thereby influencing the mRNA and protein levels. To overcome this limitation, we searched for genes, which are transcribed and translated in all three developmental stages of *L. mexicana* [22,72,73]. The *Β-TUBULIN* is such a gene. Indeed, it encodes a major structural element of microtubules, which play an important role in numerous biological processes. It is well documented that TUBULIN is expressed in all developmental stages of *L. mexicana* [74]. While the transcription is fairly stable, the protein is more abundant in the procyclic promastigotes than in amastigotes [75,76].

In *L. mexicana* MHOM/GT/2001/U1103 (genome available from the TriTrypDB [47]), Β-TUBULIN is encoded by several genes located on chromosomes 32 (2 tandemly arranged copies, annotated as *LmxM.32.0792* and *LmxM.32.0794*), 21 (a single copy *LmxM.21.1860*), and 8 (a single copy *LmxM.08.1230*). These protein coding sequences are conserved with rare amino acid (aa) substitutions, while UTRs are distinctive. These alterations in the flanking regions apparently ensure differential expression during parasite’s dixenous life cycle [77,78].

Our model organism is *L. mexicana* MNYC/BZ/1962/M379, an isolate, which is different from the TriTrypDB reference MHOM/GT/2001/U1103. We previously reported genomic sequencing of this isolate using Illumina-based methodology [79], but the repeated nature of the *TUBULIN-*encoding locus did not allow us to assemble its sequence properly. To overcome this hurdle, we used a long-read generating PacBio sequencing platform, which allowed us to assemble a 990 kb-long contig, containing over 70% of the *L. mexicana* chromosome 32 (the final assembly was 33.9 Mb, with N50 of 640 kb). We found that this contig contains seven almost identical tandemly arranged copies of *Β-TUBULIN* (GenBank Acc. No. MT431629), while the corresponding loci on chromosomes 8 and 21 contained only single *Β-TUBULIN* copies (S1 Fig). All seven copies on chromosome 32 have UTRs typical to those of *LmxM.32.0792*, while genes on chromosome 8 and 21 have different UTR sequences.

We decided to take advantage of this genomic organization and replaced an ORF for the first gene in the tandem (*LmxM.32.0792*) by the HA-tagged hygromycin B resistance gene. Western blotting analysis revealed that this gene is stably expressed throughout *L. mexicana* development (S2 Fig). To avoid introduction of exogenous UTRs into the expression cassette (so the expression would be regulated only by 5ʹ and 3ʹ UTRs of the stable expressed *LmxM.32.0792*), we used the 2A self-cleaving peptide. This is a short, 19–22 aa long sequence, which is recognized by the eukaryotic ribosome and skipped during translation. As a result, two separate proteins, located upstream and downstream of the 2A linker sequence, are produced simultaneously [80,81]. From the most widely used 2As, we chose that one derived from *Teschovirus A* because of its high cleavage efficiency [82]. Importantly, such bicistronic expression is regulated by endogenous UTRs and co-expression level of both proteins is equal. Of note, another system, which relies on a 2A peptide of *Thosea asigna virus*, has been previously reported in *L. mexicana* and used to tag endogenous proteins [83,84].

To prove the concept, we first generated the expression cassette *SAT::2A::mCHERRY* by fusing genes encoding streptothricin acetyltransferase (SAT) and fluorescent protein mCHERRY *via* a 2A peptide linker (S3 Fig). Such orientation of genes (*SAT* followed by *mCHERRY*) was chosen because 2A self-cleavage occurs at its C-terminus, leaving only a single aa at the N-terminus of a downstream protein. Replacement of a single *LmxM.32.0792* allele through homologous recombination resulted in a transgenic *L. mexicana*-mCHERRY clonal cell line (S3A Fig). As the verification of correct genome integration by PCR or Illumina whole-genome sequencing was difficult (see above), we again confirmed it by PacBio sequencing. We selected reads bearing *SAT::2A::mCHERRY* sequence and assembled them. These local assemblies produced a contig of 6,602 bp with *SAT::2A::mCHERRY* flanked by *Β-TUBULIN* gene sequences. Coverage of this contig was ~ 13*x* (approximately half of the average coverage for other loci), because only one of the two alleles was replaced. This coverage was not enough to correct all sequencing errors in the consensus sequence. Nevertheless, the presence of the *Β-TUBULIN* gene sequences and their UTRs around the *SAT::2A::mCHERRY* sequence clearly demonstrated that replacement took place in the *Β-TUBULIN* cluster on chromosome 32 (not in a single copy loci on chromosomes 8 or 21, S4 Fig). Due to the high rate of sequencing errors of PacBio technology, high sequence identity of the *Β-TUBULIN* genes within a cluster on chromosome 32, and average read length being shorter than that of the *Β-TUBULIN* cluster size, it was not possible to determine with confidence, which of the 7 copies of the gene was replaced.

The *L. mexicana*-mCHERRY cells were differentiated *in vitro* [85] and mCHERRY expression was assayed by RT-qPCR (S3B Fig) and Western blotting (S3C Fig). The differentiation was controlled by RT-qPCR analysis of the selected marker genes *PFR1D, SHERP*, and *AMASTIN* for promastigotes (both pro- and metacyclic), metacyclic promastigotes, and amastigotes, respectively [86] and compared to the wild type *L. mexicana* cells (S5 Fig, panels A and B), confirming no major defect in differentiation *in vitro*. While the *mCHERRY* RNA level was stable, we noticed a drop in mCHERRY protein level in amastigotes. Nevertheless (and as a significant improvement over previously published system [42]), this approach allowed us to express the protein of interest throughout *L. mexicana* developmental cycle. This was further confirmed by immunofluorescent microscopy, detecting fluorescent mCHERRY in axenically differentiated procyclic/metacyclic promastigotes and amastigotes (S3D Fig). Importantly, the 2A peptide-mediated cleavage appeared to be very efficient, as no higher molecular weight un-cleaved protein was detected by anti-HA Western blotting (S3C Fig).

In conclusion, the novel bicistronic expression system from the *Β-TUBULIN* endogenous locus reported here allowed us to express and follow a protein of interest during promastigote-to-amastigote development.

### *Generation of a* L. mexicana *line expressing* CATALASE

**Figure 1.**
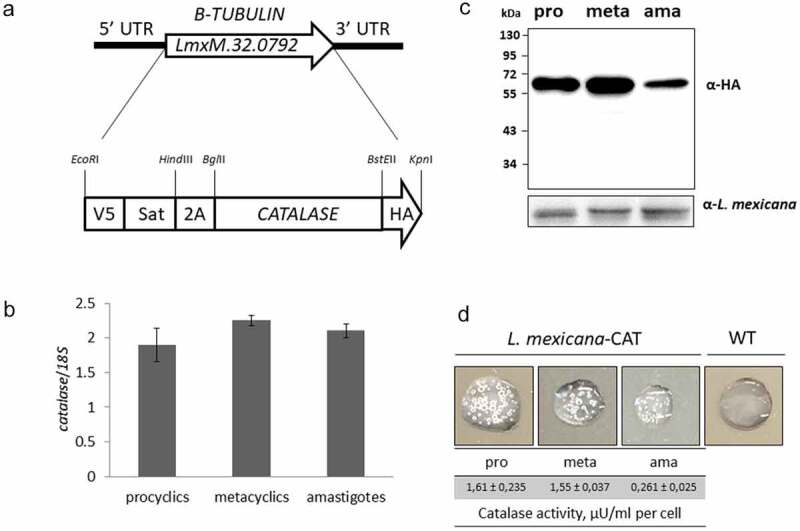
***CATALASE* expression from the *LmxM.32.0792* locus in transgenic *L. mexicana***. A) Schematic representation of the wild type and P2A-based bicistronic expression cassette for *CATALASE* integrated loci of *L. mexicana*; B) mRNA expression level of the catalase gene in axenically differentiated procyclic and metacyclic promastigotes, and amastigotes (summarized results of three independent biological replicates, normalized to expression of 18S rRNA); C) CATALASE protein expression in differentiated *L. mexicana-*CAT, analyzed by Western blotting with anti-HA-tag antibodies and anti-*Leishmania* serum as a loading control. Sizes on the left are in kD. The calculated size of V5::SAT::2A and CAT-HA are 21 and 57 kD, respectively. D) Catalase enzymatic activity (µU/ml per cell) in differentiated *L. mexicana* cells and verification of catalase activity by production of molecular oxygen (bubbles) upon addition of 3% H_2_O_2._

We next generated a *L. mexicana*-CAT (*CATALASE*) clonal line using the same approach as outlined above (Figure 1a) and verified it by PacBio sequencing (S6 Fig). The *CATALASE* used in this study originated from *Leptomonas pyrrhocoris*. Sequence analysis of this enzyme revealed conservation of the amino acids involved in heme and NADPH binding, as well as those involved in active site charge relay (S7 Fig). In contrast to their human or yeast counterpart [87,88], trypanosomatid catalases lack a peroxisomal targeting signal and localized in the cytoplasm [30,53,89].

After differentiation of the transgenic parasites *in vitro*, no major differences with respect to the differentiation marker genes *PFR1D, SHERP*, and *AMASTIN* were detected between *L. mexicana-*CAT and *L. mexicana-*mCHERRY (S5 Fig), implying that expression of catalase does not influence transcription of these genes. The mRNA level of *CATALASE* was similar in procyclic and metacyclic promastigotes, and amastigotes (Figure 1b), while its protein level was lower in amastigotes (Figure 1c). This pattern of expression was similar to that of *mCHERRY* and may reflect intrinsic properties of the *Β-TUBULIN* locus [74,76]. Similar to *L. mexicana-*mCHERRY (S3 Fig), the 2A-mediated cleavage of catalase was efficient (no visible band corresponding to the unprocessed SAT-2A-CAT protein) and the resultant expressed protein was enzymatically active in procyclic and metacyclic promastigotes, and amastigotes (Figure 1d). The calculated enzymatic activity (µU/ml per cell, Figure 1d) for differentiated parasites correlated well with Western blotting data (Figure 1c). Expression of catalase in procyclic promastigotes made cells more resistant to hydrogen peroxide (EC_50_ values for *L. mexicana*-mCHERRY and *L. mexicana*-CAT were 60.26 and 118.8 µM of H_2_O_2_, respectively) (S8 Fig). We did not observe any statistically significant difference in the rate of cell division *in vitro* between the WT, *L. mexicana*-mCHERRY, and *L. mexicana*-CAT lines (S9 Fig).

**Expression of catalase has little effect on global transcription, or expression of genes involved in oxidative stress response or heme metabolism**

Differential expression analysis was carried out for all genes, but only 30 (including tRNAs and ncRNAs) had a fold change value of >2 (BioProject PRJNA660365). Catalase is a heme-containing enzyme involved in ROS protection, thus we specifically analyzed genes involved in heme metabolism and oxidative stress response (S2 Table). Only 3 and 11 genes involved in heme metabolism and oxidative stress response pathway, respectively, were differentially expressed (highlighted in S2 Table) with adjusted *p*-values below 0.05. However, the fold change for all these transcripts was below 2.

### *Impaired development of* L. mexicana*-CAT in* Lutzomyia longipalpis

**Figure 2.**
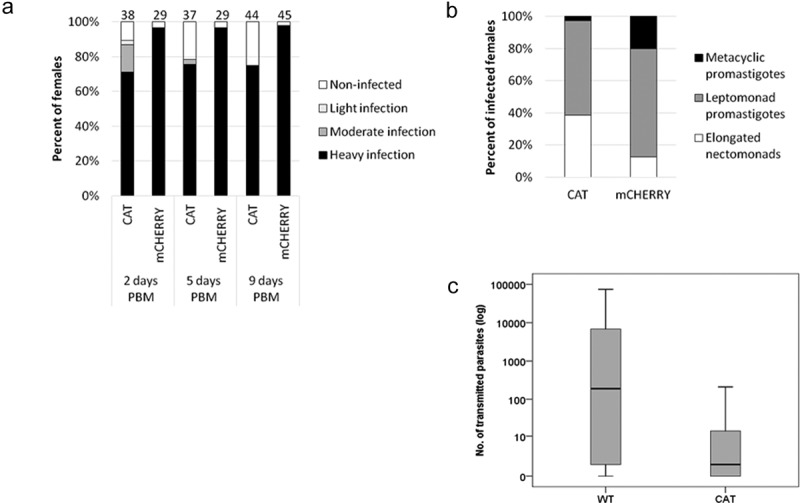
**Development of *L. mexicana*-CAT in *Lutzomyia longipalpis***. A) Rates of infections (%) in *L. longipalpis* with *mCHERRY* (mCHERRY) and *CATALASE*-expressing (CAT) *L. mexicana*. Data are summarized for four independent experiments. Numbers of dissected females are shown above the bars; B) Representation of *L. mexicana* morphological forms in guts of *L. longipalpis* on day 9 PBM. 500 parasites were measured in each group; C) Numbers of *L. mexicana* transmitted by *L. longipalpis* to BALB/c mice. Data are summarized for 17 and 21 tissue samples from the sandfly bite sites for the wild type (WT) and *L. mexicana*-CAT (CAT), respectively

In sandflies, the development of *L. mexicana*-mCHERRY and *L. mexicana*-CAT was followed on days 2, 5, and 9 post-blood meal (PBM). On day 2 PBM, heavy infections were observed in most females and parasites were localized in the endoperitrophic space, i.e. inside the bloodmeal surrounded by the peritrophic matrix. High infection rates were also documented post defecation of the blood remnants (days 3–4 PBM) and most females remained infected by day 9 PBM. At this time point, the intensity of infection was heavy and parasites were localized in the thoracic part of the midgut colonizing the stomodeal valve (Figure 2a). Females infected with *L. mexicana-*CAT, showed only slightly lower parasite burden during the early stages of infections (χ^2^ = 1.193, df = 1, p = 0.379 and χ^2^ = 4.559, df = 1, p = 0.066 on day 2 and 5 PBM, respectively). However, on day 9 PBM the percentage of infected females was about 75% for the *L. mexicana*-CAT strain, compared to 95% for the *L. mexicana*-mCHERRY (Figure 2a). This difference was statistically significant (χ^2^ = 9.895, df = 1, p = 0.002).

Morphological analysis of the infections 9 days PBM (500 promastigotes per strain) revealed that development of *L. mexicana-*CAT was considerably impaired, compared to that of *L. mexicana*-mCHERRY (Figure 2b). This defect manifested in the significantly lower representation of macrophage-infective metacyclic promastigotes (and concomitant higher representation of elongated nectomonads) in sandflies infected with *L. mexicana*-CAT (2.6% versus to 20% in control group, χ^2^ = 137.256 df = 2, p < 0.0001). Of note, a lesser impact of catalase expression on metacyclogenesis was observed *in vitro*, where percentage of metacyclic promastigotes at day 9 of differentiation were 35%, 36%, and 21% for WT, *L. mexicana*-mCHERRY, and *L. mexicana*-CAT, respectively (S9 Fig, data in brackets). No differences were documented when comparing infectivity of WT and *L. mexicana*-mCHERRY lines (χ^2^ = 0.459, df = 1, p = 1 for day 2; χ^2^ = 0.564, df = 1, p = 1 for day 5; and χ^2^ = 0.339, df = 1, p = 1 for day 9) (S10A Fig) and representation of morphological forms in guts of *L. longipalpis* on day 9 PBM (χ^2^ = 0.228, df = 2, p = 0.893) (S10B Fig), indicating that neutral (mCHERRY) genetic manipulation of the locus itself has no effect on *L. mexicana* virulence in insects. We concluded that parasites, expressing catalase, retained the ability to produce heavy infection in the natural vector *L. longipalpis* with colonization of the stomodeal valve and production of metacyclic parasites. However, the infection rate and proportion of metacyclic promastigotes in these flagellates have been significantly decreased compared to the control group *L. mexicana*-mCHERRY by day 9 PBM.

Transmission experiments demonstrated that *L. longipalpis* infected with WT and *L. mexicana*-CAT did not differ in feeding time on BALB/c mice (Table 1), while significant differences were observed in transmission efficiencies (Figure 2c). Sandfly females infected with WT and *L. mexicana*-CAT transmitted parasites to mice ears in 15/17 (88%) and 13/21 (62%) cases, respectively. Moreover, the *Leishmania* load (the number of parasites) also differed significantly. It was over 1,000 parasites (maximum 74,300) for the WT-infected females, and mostly below 100 (maximum 211) parasites in females infected with *L. mexicana*-CAT (Table 2, Figure 2c).

**Table 1.** **Feeding time of *L. longipalpis* infected with *L. mexicana* wild type parasites (WT) and *L. mexicana*-CAT (CAT)**. All time data are given in seconds. The differences between groups were verified by non-parametric Mann-Whitney *U* test

	*L. mexicana* line used for sandfly infection	
	WT	CAT
Mean (Median) feeding time	1,155 (840)	688 (450)
Std. deviation	873	519
Range (Minimum – Maximum)	188–2,460	135–1,910
The difference in median	*P* = 0.514	
The difference in distribution	*P* = 0.078

**Table 2.** **Numbers of *L. mexicana* transmitted by *L. longipalpis* to ears of BALB/c mice**. WT, wild type parasites; CAT, *L mexicana*-CAT. The differences between groups were verified by nonparametric Mann-Whitney U test

	*L. mexicana* line used for sandfly infection	
	WT	CAT
Mean (Median)	7,893 (189)	19 (1)
Std. deviation	18,165	49
Range (Minimum – Maximum)	0–74,300	0–211
The difference in median	*P* = 0.122	
The difference in distribution	*P* = 0.006	

### *Catalase compromises ability of* L. mexicana *to infect macrophages* in vitro

**Figure 3.**
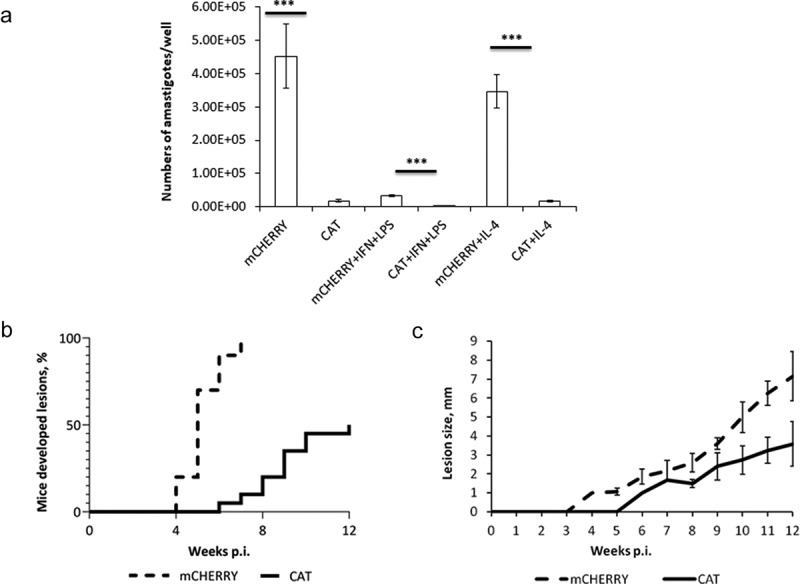
**Development of *L. mexicana*-CAT in macrophages *in vitro* and BALB/c mice *in vivo***. A) Number of amastigotes in BMMɸ of *mCHERRY* (mCHERRY) and *CATALASE*-expressing (CAT) *L. mexicana. T*-test statistical values are symbolized by asterisks: *** (p < 0.001); B) Lesions appearance and development in mice infected with *mCHERRY* (mCHERRY) and *CATALASE*-expressing (CAT) *L. mexicana*; C) Average lesion size in mm produced by *mCHERRY* (mCHERRY) and *CATALASE*-expressing (CAT) *L. mexicana* in BALB/c mice. Average and standard deviation was calculated only for mice developed lesions

Next, we tested the ability of *L. mexicana*-CAT to establish infection in mouse bone marrow-derived macrophages (BMMɸ). After 72 hours p.i. of macrophages with metacyclic promastigotes, the number of *L. mexicana*-CAT amastigotes was significantly lower as compared with *Leishmania*-mCHERRY parasites in unstimulated (p < 0.001), as well as classically- (p < 0.001) and alternatively- (p < 0.001) stimulated macrophages (Figure 3a).

The observed differences in the number of amastigotes in unstimulated and classically stimulated macrophages were expected, as stimulation with IFN-γ + LPS generally leads to a considerable reduction in parasite load. The numbers of *Leishmania*-mCHERRY and *L. mexicana-*CAT amastigotes were reduced in macrophages, stimulated with IFN-γ + LPS (p < 0.001 for both *Leishmania* lines). Of note, no statistically significant difference in infection of macrophages was noted between WT and *L. mexicana*-mCHERRY lines (p = 0.535; 0.0859; and 0.303 for unstimulated, classically, and alternatively stimulated macrophages) (S11 Fig). No differences in NO production were observed for unstimulated and alternatively stimulated macrophages in both WT (p = 0.103) and CAT (p = 0.91) groups (S12 Fig), ruling out the potential role of NO in the documented phenotypic manifestation.

No extracellular parasites were detected 2 hours after infection that makes it reasonable to assume that defect in the ability of the *L. mexicana*-CAT cells to infect macrophages *in vitro* was not associated with their initial uptake.

### *Decreased virulence of* L. mexicana*-CAT in mice*

We also tested *L. mexicana*-CAT infectivity in a murine model and documented a clear difference in lesions development between the *mCHERRY* and *CATALASE*-expressing *L. mexicana*. Lesions appeared in all animals infected with *L. mexicana*-mCHERRY, while only in 10 out of 20 in the CAT group. In addition, lesions produced by *L. mexicana*-CAT appeared 2 weeks later and developed slower than in the control group (p < 0.001) (Figure 3b and c). Of note, no statistically significant difference was observed in lesions development between the WT and *mCHERRY*-expressing *L. mexicana* (p = 0.547) (S11 Fig).

To assess the transmission-to-sandfly (xenodiagnosis) potential of *Leishmania*-CAT parasites we allowed uninfected flies to feed on the lesions of infected mice by week 12 p.i. The results of these experiments are summarized in Table 3. All 10 mice infected with WT parasites were infectious to sandflies, and (as an average of two experiments) 28.5% of sandflies have acquired parasites. In sharp contrast, only 3 out of 10 animals were infectious to sandflies in the CAT group with only 4% of sandflies acquiring *Leishmania*.

**Table 3.** Xenodiagnostic experiments performed by week 12 p.i. *L. longipalpis* females were allowed to feed on the inoculated ears of anaesthetized BALB/c mice

*L. mexicana* strain	Experiment No.	Mouse	Infected/Total No. of sandflies	% of infected sandflies
WT	1	1	2/20	10.0
		2	4/17	23.6
		3	3/21	14.3
		4	4/15	26.7
		5	6/19	31.6
		Total	19/92	20.6
	2	1	10/36	27.8
		2	7/37	18.9
		3	5/37	13.5
		4	17/40	42.5
		5	20/32	62.5
		Total	59/182	32.4
		Total 2 exp.	78/274	28.5
CAT	1	1	3/10	30.0
		2	3/9	33.3
		3	0/6	0
		4	1/17	5.9
		5	0/9	0
		Total	7/51	13.7
	2	1	0/15	0
		2	0/27	0
		3	0/30	0
		4	0/24	0
		5	0/28	0
		Total	0/124	0
		Total 2 exp.	7/175	4.0

In addition, we also confirmed that *CATALASE* was retained by *L. mexicana* and expressed after passaging through sandflies and mice, ruling out the possibility that parasites that no longer express catalase had selection advantage *in vivo* (S13 Fig). Interestingly, its expression level was lowered compared to that of *in vitro* cultivated promastigotes, again supporting our argument that presence of catalase is detrimental for *Leishmania in vivo.*

## Discussion

One of the core questions of evolutionary biology is the origin of parasitism. The transition from a free living to a parasitic life style was accompanied by the gain and loss of multiple genes (often whole protein gene families) in order to adapt to the new environment within the host [90]. Understanding this process requires to compare the free-living and parasitic species [91]. In this respect, kinetoplastids are an ideal model group because they unite obligatory parasitic trypanosomatids and their free-living relatives, bodonids [32,92]. A large number of kinetoplastid genomes have been sequenced and analyzed [2], facilitating our understanding of the basic differences between these groups of organisms. The comparative analyses of the dixenous and monoxenous parasites illuminate the key features, which have allowed the former to adapt to the environment of vertebrate hosts [22,93,94].

In terms of the complex parasitic lifestyle, a recently established subfamily Leishmaniinae is particularly interesting because it encompasses phylogenetically related monoxenous and dixenous trypanosomatids [95,96]. Interestingly, the catalase gene was secondarily lost from all dixenous members of the Leishmaniinae [22,26,32]. The fact that hydrogen peroxide has been proposed as a signaling molecule in *Leishmania* amastigote differentiation [28,29] prompted us to hypothesize that catalase may be not compatible with the dixenous life cycle of *Leishmania* spp. To investigate this, we expressed the enzyme from the monoxenous *L. pyrrhocoris* in dixenous *L. mexicana*. In order to do that we had to circumvent the methodological difficulty of expressing a protein throughout the developmental cycle of *L. mexicana*. Numerous previous attempts failed, mainly because all routinely used genetic manipulation techniques in *Leishmania* rely on the incorporation of exogenous UTRs. These genetic elements greatly impact on the expression of transgenic DNA sequences [42,97,98]. In this work, we have solved this issue by using the stably expressed *LmxM.32.0792 Β-TUBULIN* locus and a modified genetic system, based on the self-cleaving 2A peptide. This approach has rendered unnecessary the requirement to use exogenous UTRs. The choice of the locus was primarily determined by its stable transcription and translation, yet, in retrospect, the multiple copies of *Β-TUBULIN* gene made it more complicated than expected [77]. The 2A self-cleaving peptide was discovered 30 years ago [99] and since then it has been used in many species, including zebrafish, mice, silkworm, and algae [82,100]. In this work, the use of the P2A self-cleaving peptide in *L. mexicana* resulted in efficient expression of both the antibiotic resistance and catalase genes, integrated into the *Β-TUBULIN* locus.

We hypothesized that the loss of the catalase gene was a prerequisite for the dixeny in *Leishmania* [26]. It has been indirectly supported by studies in other dixenous trypanosomatids, *T. brucei* and *T. cruzi*, where catalase was found to have a disadvantageous effect on their vitality *in vivo* [30,31]. To investigate this in detail, we established a *L. mexicana* line that expressed catalase during differentiation (Figure 1), and explored the role of the enzyme *in vitro* and *in vivo*, both in sandflies and mouse models of infection.

In insects, the production of ROS is important for innate immune defense and regulation of insect gut-microbe homeostasis [101]. In this respect, the loss of catalase appears to be disadvantageous. However, ROS production in *L. longipalpis* does not change significantly upon *L. mexicana* infection [102,103]. This implies that H_2_O_2_ level is remained low, allowing parasites to thrive and making catalase redundant for the insect life cycle stages of *Leishmania*. In this work, we demonstrated that even though the *Leishmania*-CAT strain showed only slightly reduced infectivity compared to the control *Leishmania*-mCHERRY group, its metacyclogenesis was significantly impaired (Figure 2). The reduction in percentage of metacyclic promastigotes was accompanied by a concomitant increase in the proportion of elongated nectomonads. The latter are the first to reach the ectoperitrophic space and, therefore, the first to register ROS, including H_2_O_2_ [7]. As such, the proportional distribution of elongated nectomonads, leptomonads, and metacyclic promastigotes in the insects, infected by *L. mexicana*-CAT, is consistent with the hypothesis that H_2_O_2_ is a signaling molecule in *Leishmania* differentiation. Catalase inhibits metacyclogenesis and, consequently, the virulence of *Leishmania* [104]. Expectedly, the transmission efficiency of *L. mexicana*-CAT was dramatically lower than that of the wild type, emphasizing the need of metacyclic promastigotes for mammalian host infection.

Macrophages infected with *L. mexicana*-CAT showed lower amastigote burdens compared to those infected with *Leishmania*-mCHERRY parasites. Metacyclics of both *L. major* and *L. mexicana* are not as sensitive to H_2_O_2_ as procyclic promastigotes. Amastigotes survival in phagolysosome is primarily determined by host’s intracellular level of ROS [105]. In mice, lesions induced by *L. mexicana*-CAT appeared dramatically slower than those caused by *Leishmania*-mCHERRY parasites (Figure 3). It was recently demonstrated that infection of mice with a mix of metacyclic promastigotes and non-infective nectomonads of *L. mexicana* results in a faster disease progression and larger lesions compared to those induced only by a high dose of metacyclics [106]. This implies that metacyclic promastigotes *per se* are not the sole determinant of the infection outcome, and that other stimuli (e.g. peroxide) are required for proper amastigote differentiation. Xenodiagnostic experiments demonstrated that parasites in lesions, initiated by *L. mexicana*-CAT, were less transmittable to the sandflies than those from the lesions of wild type *L. mexicana.*

In summary, the presence of catalase significantly reduces *L. mexicana* differentiation in sandflies, transmission potential, and virulence in the mammalian hosts. We conclude that the presence of catalase is not compatible with the dixenous life cycle of this parasite.

## Supplementary Material

Supplemental Material

## Data Availability

The data that support the findings of this study are openly available in NCBI BioProject at https://www.ncbi.nlm.nih.gov/bioproject/, reference number BioProject PRJNA660365.
