# Supplementary material for: Catalase impairs Leishmania mexicana development and virulence
Source: Virulence. 2021 Mar 16;12(1):852–67. doi: 10.1080/21505594.2021.1896830 (PMC7971327; doi:10.1080/21505594.2021.1896830)
Supplement: Supplemental Material [file KVIR_A_1896830_SM4516.zip › S03 Fig R2.pptx]

## Slide 1
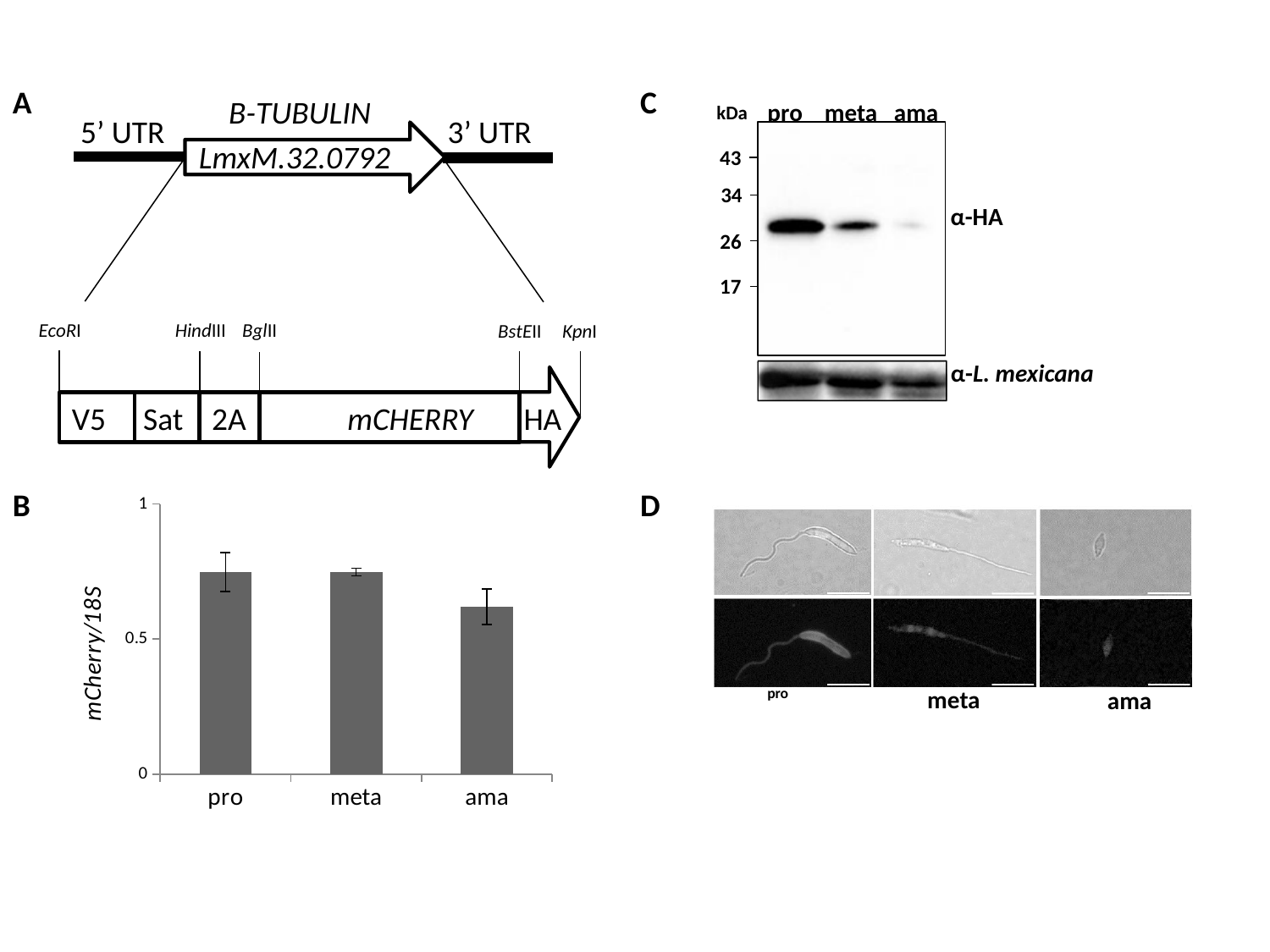

C
A
Β-TUBULIN
5’ UTR
3’ UTR
LmxM.32.0792
EcoRI
HindIII
BglII
BstEII
KpnI
V5 Sat 2A mCHERRY HA
pro meta ama
kDa
43
34
α-HA
26
17
α-L. mexicana
B
D
### Chart
| Category | |
|---|---|
| pro | 0.7480831688658115 |
| meta | 0.7480945049521859 |
| ama | 0.6193141962660692 |
pro
meta
ama
