# Supplementary material for: Catalase impairs Leishmania mexicana development and virulence
Source: Virulence. 2021 Mar 16;12(1):852–67. doi: 10.1080/21505594.2021.1896830 (PMC7971327; doi:10.1080/21505594.2021.1896830)
Supplement: Supplemental Material [file KVIR_A_1896830_SM4516.zip › S05 Fig R2.pptx]

## Slide 1
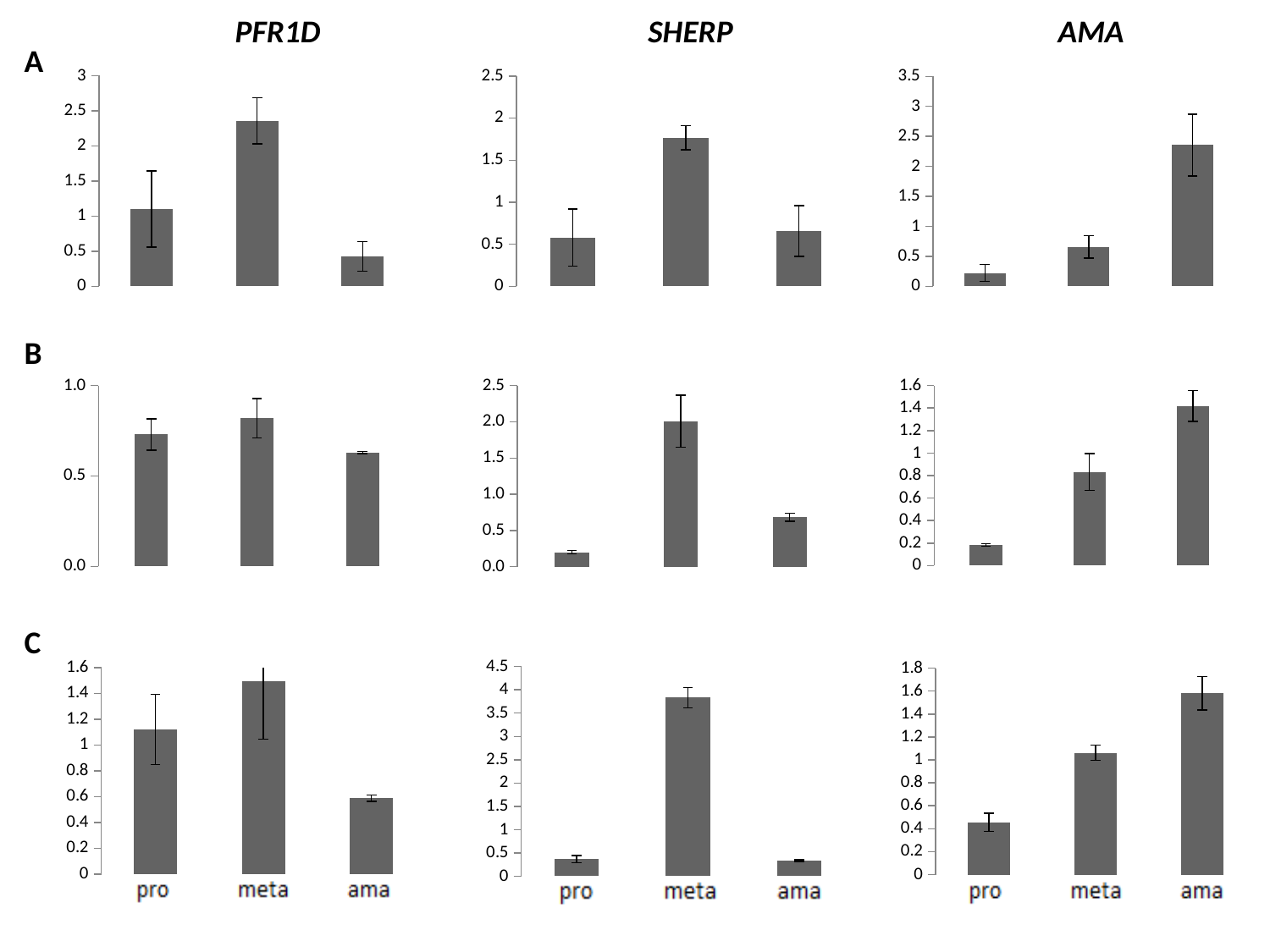

PFR1D
SHERP
AMA
A
### Chart
| Category | Wt |
|---|---|
| Pro | 0.2236702098418669 |
| Meta | 0.6571937827805197 |
| Ama | 2.3543182168257015 |
### Chart
| Category | Wt |
|---|---|
| Pro | 1.1011135210085568 |
| Meta | 2.359318659220172 |
| Ama | 0.42480548211218994 |
### Chart
| Category | Wt |
|---|---|
| Pro | 0.5795052681149032 |
| Meta | 1.7677960611289463 |
| Ama | 0.6566625492234841 |B
### Chart
| Category | |
|---|---|
| pro | 0.2 |
| meta | 2.01 |
| ama | 0.68 |
### Chart
| Category | |
|---|---|
| pro | 0.18521505207974895 |
| meta | 0.8322534203595083 |
| ama | 1.4190094291740079 |
### Chart
| Category | |
|---|---|
| pro | 0.7300000000000006 |
| meta | 0.8200000000000006 |
| ama | 0.6300000000000009 |C
### Chart
| Category | |
|---|---|
| pro | 0.45604319860231696 |
| meta | 1.0628462151075941 |
| ama | 1.5798940246793542 |
### Chart
| Category | |
|---|---|
| pro | 1.1198915775596674 |
| meta | 1.4926147569546218 |
| ama | 0.5881755170788152 |
### Chart
| Category | |
|---|---|
| pro | 0.36450171752936616 |
| meta | 3.8334921064336833 |
| ama | 0.33802463689940093 |
