# Supplementary material for: Catalase impairs Leishmania mexicana development and virulence
Source: Virulence. 2021 Mar 16;12(1):852–67. doi: 10.1080/21505594.2021.1896830 (PMC7971327; doi:10.1080/21505594.2021.1896830)
Supplement: Supplemental Material [file KVIR_A_1896830_SM4516.zip › S06 Fig R2.pptx]

## Slide 1
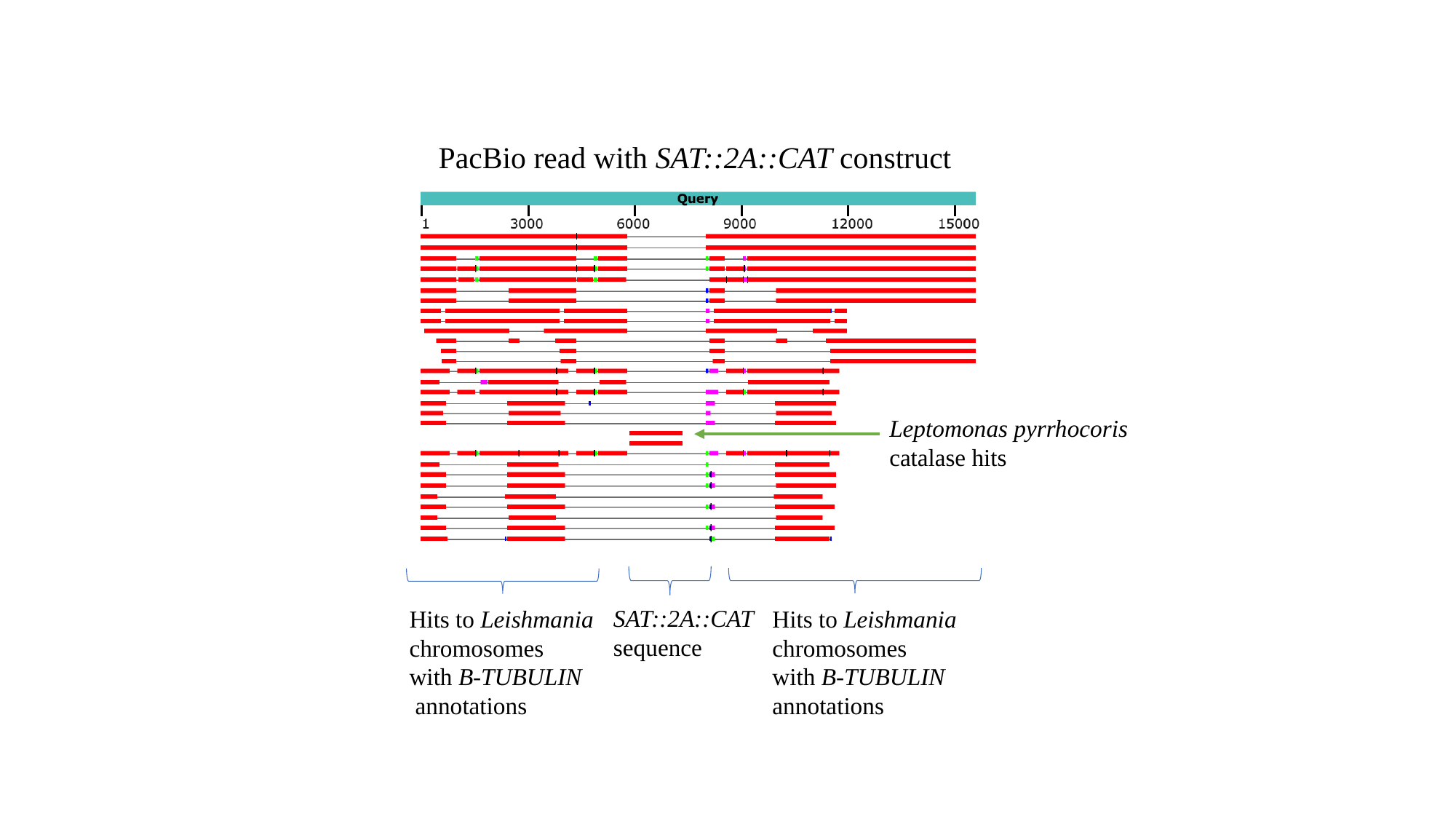

PacBio read with SAT::2A::CAT construct
Leptomonas pyrrhocoris
catalase hits
SAT::2A::CAT
sequence
Hits to Leishmania chromosomes
with Β-TUBULIN
 annotations
Hits to Leishmania chromosomes
with Β-TUBULIN annotations
