# Supplementary material for: Catalase impairs Leishmania mexicana development and virulence
Source: Virulence. 2021 Mar 16;12(1):852–67. doi: 10.1080/21505594.2021.1896830 (PMC7971327; doi:10.1080/21505594.2021.1896830)
Supplement: Supplemental Material [file KVIR_A_1896830_SM4516.zip › S07 Fig R2.rtf]

                                 10        20        30        40        50        60        70        80        90       100                  
                        ....|....|....|....|....|....|....|....|....|....|....|....|....|....|....|....|....|....|....|....|
Homo sapiens            MADSRDPASDQMQHWKEQRAAQKADV-LTTGAGNPVGDKLNVITVGPRGPLLVQDVVFTDEMAHFDRERIPERVVHAKGAGAFGYFEVTHDITKYSKAKV 
Leptomonas pyrrhocoris  .PSNES..V-----------.ACPMR-...EF.A..ANNDDMM.A.R...A.L...WLLEKL......E....RM....S....T.T......Q.TR..L 
Leptomonas seymouri     .LSNES.SV-----------.ACPMR-...EF.A.LVNND.TM.A.....T.L...WLLEKL......E....RM....T....T.T..N.....T...L 
Crithidia fasciculata   .PSNES.SV-----------GVCPMR-...EF.A..VNND.TA.A.R...T.L...WLLEKL..L...E....RM....S....T.T......Q.T...I 
Crithidia thermophila   .QTQSNA.AA.------.P-.VCPMRF...EF.A..VNNDDTA.A.R...T.L...WLLEKL......E.A..RM....T.V..T.T......A.T...M 

                                110       120       130       140       150       160       170       180       190       200         
                        ....|....|....|....|....|....|....|....|....|....|....|....|....|....|....|....|....|....|....|....|
Homo sapiens            FEHIGKKTPIAVRFSTVAGESGSADTVRDPRGFAVKFYTEDGNWDLVGNNTPIFFIRDPILFPSFIHSQKRNPQTHLKDPDMVWDFWSLRPESLHQVSFL 
Leptomonas pyrrhocoris  .SEV....DMF.........K.AP.LE..I....M.....E...........V.YF..ALR..DLN.AV..D.K.NMRSAQNK...FTML..A....TID 
Leptomonas seymouri     .SEV..Q.ELF.........R.AP.LD..I..........E...........V.YF...LH..DLN.AV..D.R.NMRSAQHK...FT.L.......TID 
Crithidia fasciculata   .SEV....DMF.........K.AP.LD..I....M.....E....M......V.YF..ALR..DLN.AV..D.K.NMRN.QSK...FT.L..A....TID 
Crithidia thermophila   .SEV....DCF.........R.AP.LD..I....M.....E...........V.YF..GLR..DLN.AV..D.K.NMRS.QAK...FTSL..A....TID 

                                210       220       230       240       250       260       270       280       290       300         
                        ....|....|....|....|....|....|....|....|....|....|....|....|....|....|....|....|....|....|....|....|
Homo sapiens            FSDRGIPDGHRHMNGYGSHTFKLVNANGEAVYCKFHYKTDQGIKNLSVEDAARLSQEDPDYGIRDLFNAIATGKYPSWTFYIQVMTFNQAETFPFNPFDL 
Leptomonas pyrrhocoris  M....L.ANY...H.F....YSFI..KN.L.WV...F.CQ.....VTDAE..QIVGT.TESSQ...VD..ER.D..R.DMKV.L..RE..KQC....... 
Leptomonas seymouri     M....L.ASY...H.FS..AYSFI..AN.L.WV...L.SQ.....VTDAE..LIVGS.TES.Q...VD..DR.D..R.DMKV.L..QD..RQC....... 
Crithidia fasciculata   M....M.ANY...H.FS..AYSFI..KD.L.WV...F.SQ......TDAE..AIVGQ.TESSQ...VD..ER.D..R.DMKV.L..QE..KQC....... 
Crithidia thermophila   M....M.VSY.Y.H.F.....S.I..KN.LIWV...F.SQ.....YTDAEVEQIVGK.TETSQ..MCD..ER.DF.R.DMK..L..QE..KQC....... 

                                310       320       330       340       350       360       370       380       390       400         
                        ....|....|....|....|....|....|....|....|....|....|....|....|....|....|....|....|....|....|....|....|
Homo sapiens            TKVWPHKDYPLIPVGKLVLNRNPVNYFAEVEQIAFDPSNMPPGIEASPDKMLQGRLFAYPDTHRHRLGPNYLHIPVNCPYRARVANYQRDGPMCMQDNQG 
Leptomonas pyrrhocoris  ..T.SQ......D..VME...V.E....D...A..S.AVVV...SF...R...A...S.G.AQ.Y...V..AS....A.-.CPFHS.H.N.L.RVDG.N. 
Leptomonas seymouri     ..T.SQ.E....D..VME...I.E....D...A..S.ALVV...SF...R...A...S.G.AQ.Y...V..SS....A.-.CP.HC.H.N.L.RVDG.N. 
Crithidia fasciculata   ..T.SQ.T....D..VME...I.E....D...A..S..VVV...SF...R...A...S.G.AQ.Y...V..SQ....A.-.CPFHT.H.N.T.RVDG.N. 
Crithidia thermophila   ..T.SQ......D..VME...I.E....D...A..S..VVV...SF...R...A...S.G.AQ.Y...V..SS....A.-.CPFHT.H.N.A.RVDG.N. 

                                410       420       430       440       450       460       470       480       490       500         
                        ....|....|....|....|....|....|....|....|....|....|....|....|....|....|....|....|....|....|....|....|
Homo sapiens            GAPNYYPNSFGAPEQQPSALEHSIQYSGEVRR--FNTANDDNVTQVRAFYVNVLNEEQRKRLCENIAGHLKDAQIFIQKKAVKNFTEVHPDYGSHIQALL 
Leptomonas pyrrhocoris  ANLQ.A...A.EWQSNAA.E.PALPVE.YACHYD.HEDD..YYSCP..LF-ELMSP..KTV.F..T.RAMQGTTKEV.LRHIH.CMKA.R...LGVAKA. 
Leptomonas seymouri     SKLQ.A...S.EWLNSAKSPVPEQPVE.FAAHYD.RKDD..YYSCP..LF-ELMRP..KTV.F..T.RAMQGTKKEV.LRHIR.CMKA.R...LGVAKA. 
Crithidia fasciculata   ANLQ.A...ANEWRSSAT.P.PVLPVE.FAAHYD.HEDD..YYSNP..LF-E.MSP..KTV.F..TGRAMDGTSKEVRLRH.R.CMKA.R...LGVAKA. 
Crithidia thermophila   ANIQ.....V.EWRSSAGSA.PTLPIE.FAGHYD.HEDD..YYSNP..LF-ELMKPDEKT..F..T.RAMQGTSKEV.LRHIQ.CMKA.K...MGVAKA. 

                                510       520       530  
                        ....|....|....|....|....|....|
Homo sapiens            DKYNAEKPKNAIHTFVQSGSHLAAREKANL 
Leptomonas pyrrhocoris  NVSE..I----------------------- 
Leptomonas seymouri     GISE..IWP--------------------- 
Crithidia fasciculata   GISES.I----------------------- 
Crithidia thermophila   GVSES.L----------------------- 
