# Supplementary material for: Catalase impairs Leishmania mexicana development and virulence
Source: Virulence. 2021 Mar 16;12(1):852–67. doi: 10.1080/21505594.2021.1896830 (PMC7971327; doi:10.1080/21505594.2021.1896830)
Supplement: Supplemental Material [file KVIR_A_1896830_SM4516.zip › S10 Fig R2.pptx]

## Slide 1
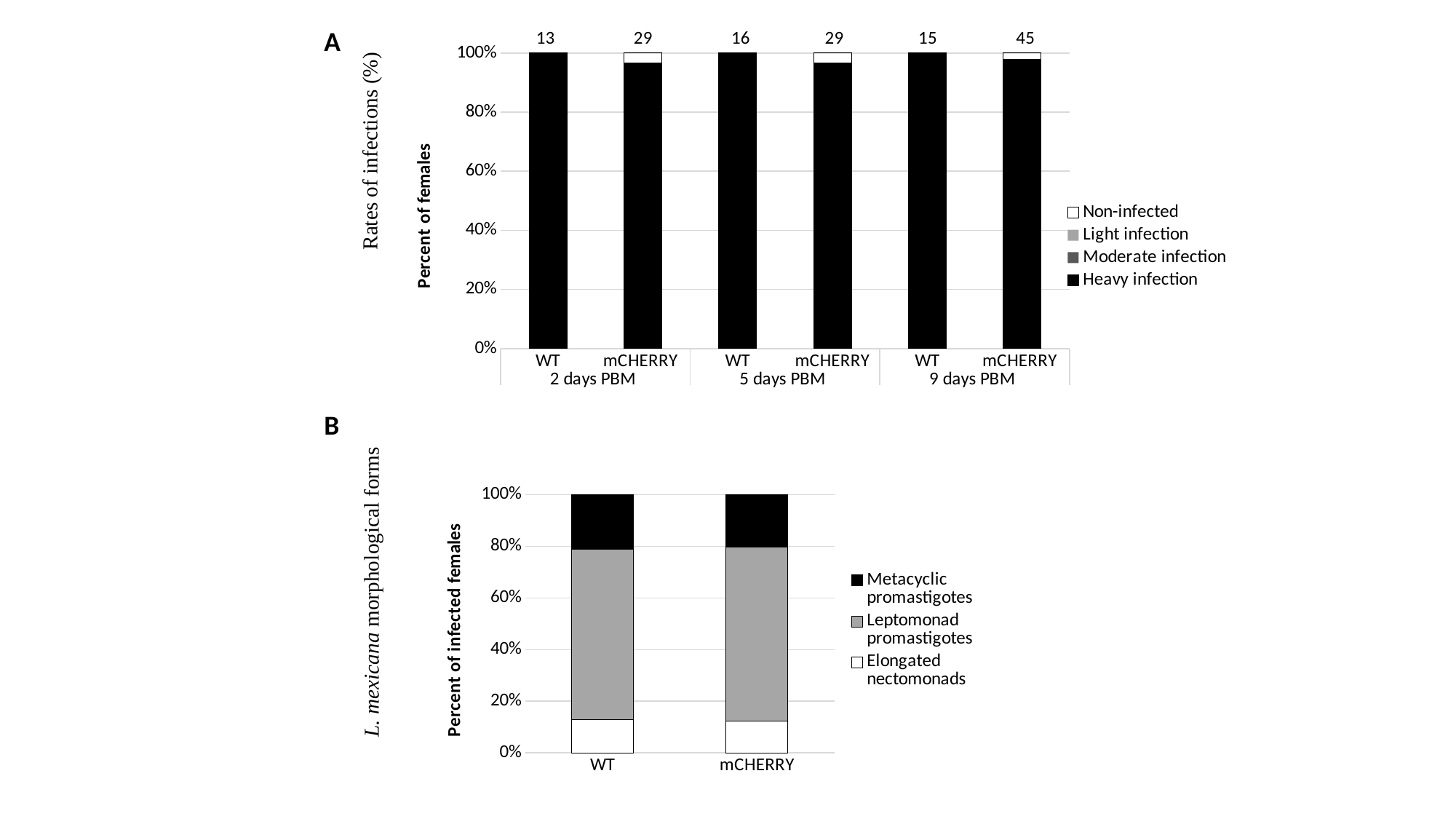

A
13 29 16 29 15 45
### Chart
| Category | Heavy infection | Moderate infection | Light infection | Non-infected |
|---|---|---|---|---|
| WT | 13.0 | 0.0 | 0.0 | 0.0 |
| mCHERRY | 28.0 | 0.0 | 0.0 | 1.0 |
| WT | 16.0 | 0.0 | 0.0 | 0.0 |
| mCHERRY | 28.0 | 0.0 | 0.0 | 1.0 |
| WT | 15.0 | 0.0 | 0.0 | 0.0 |
| mCHERRY | 44.0 | 0.0 | 0.0 | 1.0 |Rates of infections (%)
B
### Chart
| Category | Elongated nectomonads | Leptomonad promastigotes | Metacyclic promastigotes |
|---|---|---|---|
| WT | 64.0 | 330.0 | 106.0 |
| mCHERRY | 62.0 | 337.0 | 101.0 |L. mexicana morphological forms
