# Supplementary material for: Catalase impairs Leishmania mexicana development and virulence
Source: Virulence. 2021 Mar 16;12(1):852–67. doi: 10.1080/21505594.2021.1896830 (PMC7971327; doi:10.1080/21505594.2021.1896830)
Supplement: Supplemental Material [file KVIR_A_1896830_SM4516.zip › supp.legend.rtf]

Supplementary figure legends 
S1 Fig. Locations of Â-TUBULIN gene in assembled Leishmania mexicana contigs. Summary table showing gene start, end, and orientation of genes. Box colors indicate homology of assembled contigs with chromosomes from L. mexicana genome assembly from TriTrypDB (v. 2016-05-28). 
S2 Fig. Replacement of LmxM.32.0792 Â-TUBULIN gene by HYG-HA. A) Schematic representation of wild type and modified loci of L. mexicana; B) Western blotting analysis of protein expression in axenically differentiated L. mexicana cells. Whole cell extracts were probed with anti-HA monoclonal antibodies and anti-Leishmania serum (as a loading control). Sizes on the left are in kD. The calculated size of Hyg-HA is 39 kD. Abbreviations pro, meta, and ama indicate procyclic and metacyclic promastigotes, and amastigotes, respectively.  
S3 Fig. Transgenic mCHERRY expression from the Â-TUBULIN locus (LmxM.32.0792) of L. mexicana. A) Schematic representation of the wild type and P2A-based bicistronic expression cassette for mCHERRY integrated loci of L. mexicana; B) mRNA expression level of mCHERRY in axenically differentiated procyclic (pro), metacyclic (meta) promastigotes and amastigotes (ama) (summarized results of three independent biological replicates, normalized to expression of 18S rRNA); C) mCHERRY protein expression in differentiated L. mexicana-mCHERRY cells analyzed by Western blotting with anti-HA-tag antibodies and anti-Leishmania serum as a loading control. Sizes on the left are in kD. The calculated size of V5x3::SAT::2A and mCHERRY-HA are 27 and 29 kD, respectively; D) Confirmation of mCHERRY protein expression by fluorescence microscopy. Scale bars are 10 ìm. 
S4 Fig. mCHERRY in the Â-TUBULIN locus on chromosome 32. Alignment of the Â-TUBULIN UTR from the 6,602 bp contig with SAT::2A::mCHERRY insert with UTR of LmxM.32.0792 (top). Graphical representation of BLASTn hits from NCBI blast server (NCBI non-redundant nucleotide database with restriction by taxid: 5654 (Trypanosomatidae) for the 6,602 bp contig with SAT::2A::mCHERRY sequence as a query. Search was done with "word size" set to 7. The Bit scores of alignments are shown in red (over 200) and magenta (80 – 200). 
S5 Fig. Expression of Leishmania differentiation marker genes in the WT L. mexicana (A), L. mexicana-mCHERRY (B), and L. mexicana-CAT (C). RT-qPCR analysis of PFR1D (marker of pro- and metacyclic promastigotes), SHERP (marker of metacyclic promastigotes) and AMASTIN (marker of amastigotes). Data of three independent biological replicates, normalized to expression of 18S rRNA, are shown. 
S6 Fig. CATALASE in the Â-TUBULIN locus on chromosome 32. Graphical representation of BLASTn hits from NCBI blast server (NCBI non-redundant nucleotide database with restriction by taxid: 5654 (Trypanosomatidae) for the 6,602 bp contig with SAT::2A::CAT sequence as a query. Search was done with "word size" set to 7. The Bit scores of alignments are shown in red (over 200), magenta (80 – 200), green (50 – 80), and blue (40 – 50). 
S7 Fig. Alignment of amino acid sequences of selected trypanosomatid catalases with that from H. sapiens. Predicted functional domains and sites: ♦ –heme binding site, ● – residues involved in the active site charge relay, green box –NADPH binding site, blue box – tetramer interface, red box – peroxisomal targeting domain. 
S8 Fig. CATALASE increases resistance to H2O2. Sensitivity of mCHERRY and CATALASE-expressing L. mexicana procyclic promastigotes to different concentrations of H2O2. The proliferation of cells and EC50 values were quantitatively measured by Alamar blue assay. The data represent the mean values of measures ± standard deviations of three independent experiments.
S9 Fig. In vitro growth of L. mexicana-CAT, L.mexicana-mCHERRY and WT promastigotes. The initial cell density was 5 × 10^5^ cells/ml, and cultures were sub-passaged on day 3 into the media with pH 5.5 to initiate metacyclogenesis. Wild type and L. mexicana-CAT are represented by solid and–dashed lines, respectively. L. mexicana-mCHERRY graph is marked by red triangles. Averages and standard deviations were calculated based on 3 independent biological replicates. The percentage of metacyclic promastigotes at day 9 are indicated in brackets. A total of 450 parasites were measured in each group.
S10 Fig. Incorporation of mCHERRY has no effect on infectivity in insects. A) Rates of infections (%) in L. longipalpis with wild type (WT) and mCHERRY-expressing (mCHERRY) L. mexicana on days 2, 5, and 9 PBM. Numbers of dissected females were 13, 29 for WT and L. mexicana-mCHERRY, respectively, on day 2 PBM; 16, 29, respectively, on day 5 PBM; and 15, 45, respectively, on day 9 PBM; B) Representation of L. mexicana morphological forms in guts of L. longipalpis on day 9 PBM. 500 parasites were measured in each group. 
S11 Fig. Development of L. mexicana-mCHERRY in macrophages in vitro and BALB/c mice in vivo. A) Number of amastigotes in BMMɸ of the wild type (WT) and mCHERRY-expressing (mCHERRY) L. mexicana; B) Lesions appearance and development in mice infected with the wild type (WT) and L. mexicana-mCHERRY (mCHERRY); C) Average lesion size in mm produced by wild type (WT) and L. mexicana-mCHERRY in BALB/c mice. The observed differences are not statistically significant (p = 0.547). 
S12 Fig. Production of NO in macrophages infected with WT and L. mexicana-CAT parasites. N.S. = non-stimulated macrophages. 
S13 Fig. Expression of CATALASE after passaging L. mexicana-CAT in vivo in sandflies and mice. Data of three independent biological replicates (for in vivo, independent isolates from mice and sandflies) normalized to expression of 18S rRNA are shown.

Supplementary table legend 
S1 Table. List of primers used in this study. 
S2 Table. Differential expression analysis of L. mexicana-CAT and WT focused on genes involved in heme metabolism and oxidative stress response. Differentially expressed genes with adjusted p-values below 0.05 are highlighted. Gene annotations were taken from TriTrypDB.
