# Supplementary figures and images for: Catalase impairs Leishmania mexicana development and virulence
Source: Virulence. 2021 Mar 16;12(1):852–67. doi: 10.1080/21505594.2021.1896830 (PMC7971327; doi:10.1080/21505594.2021.1896830)

## Slide 1
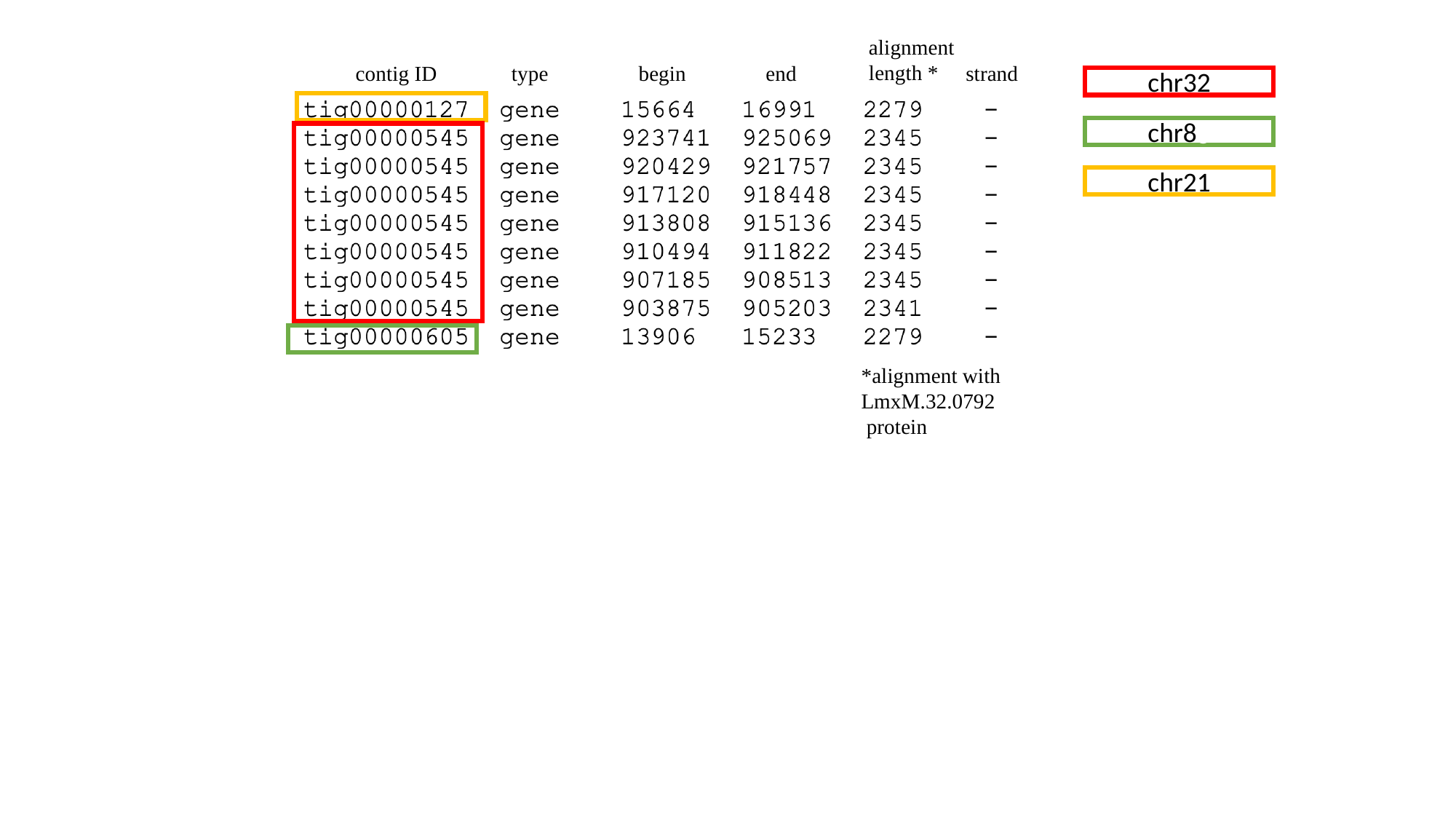

alignment
length *
contig ID
type
begin
end
strand
chr32
chr8§
chr21
*alignment with
LmxM.32.0792
 protein

Supplement: Supplemental Material [file KVIR_A_1896830_SM4516.zip › S01 Fig R2.pptx]

## Slide 1
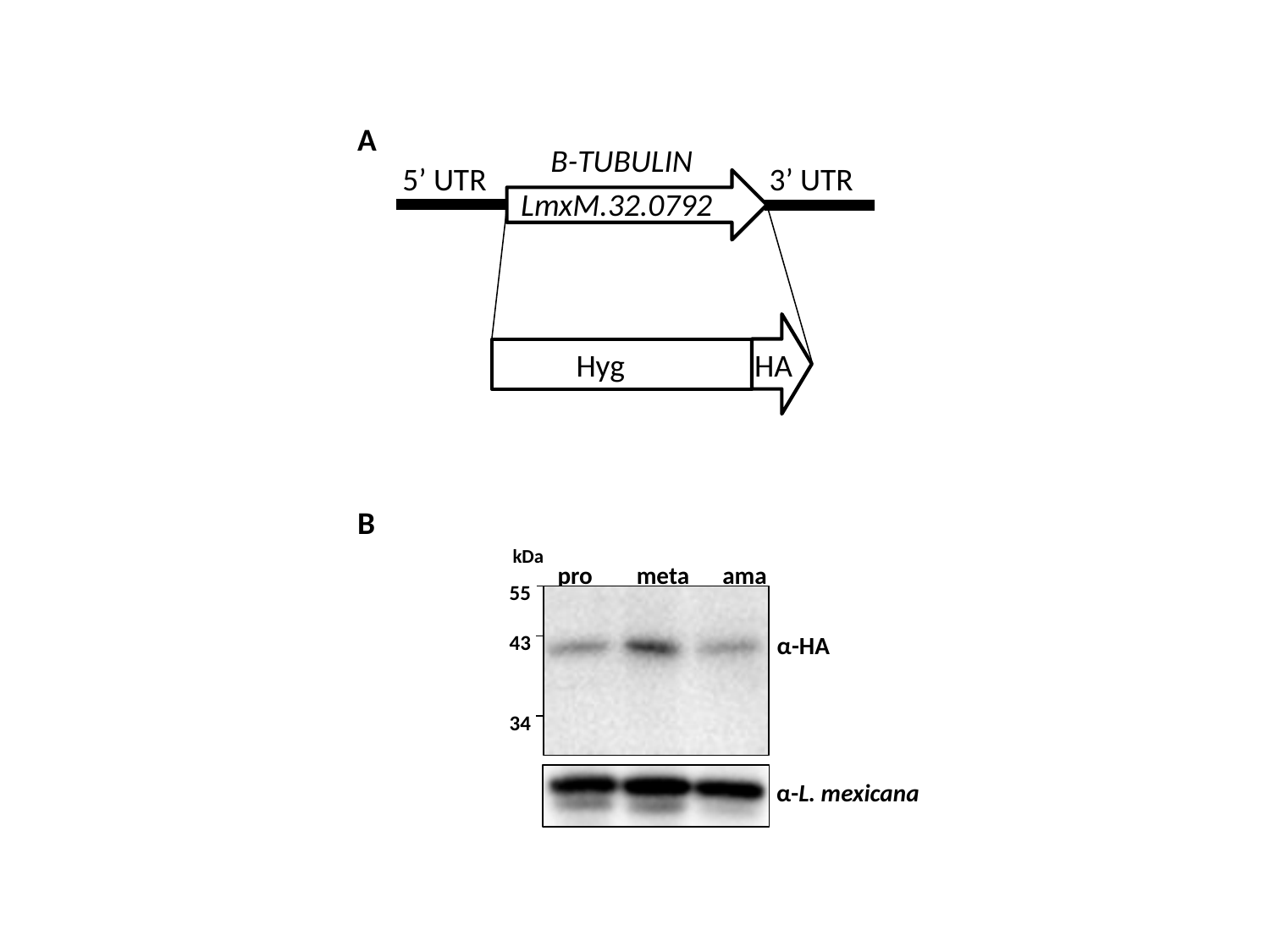

A
Β-TUBULIN
5’ UTR
3’ UTR
LmxM.32.0792
 Hyg HA
B
kDa
pro meta ama
α-HA
α-L. mexicana
55
43
34

Supplement: Supplemental Material [file KVIR_A_1896830_SM4516.zip › S02 Fig R2.pptx]

## Slide 1
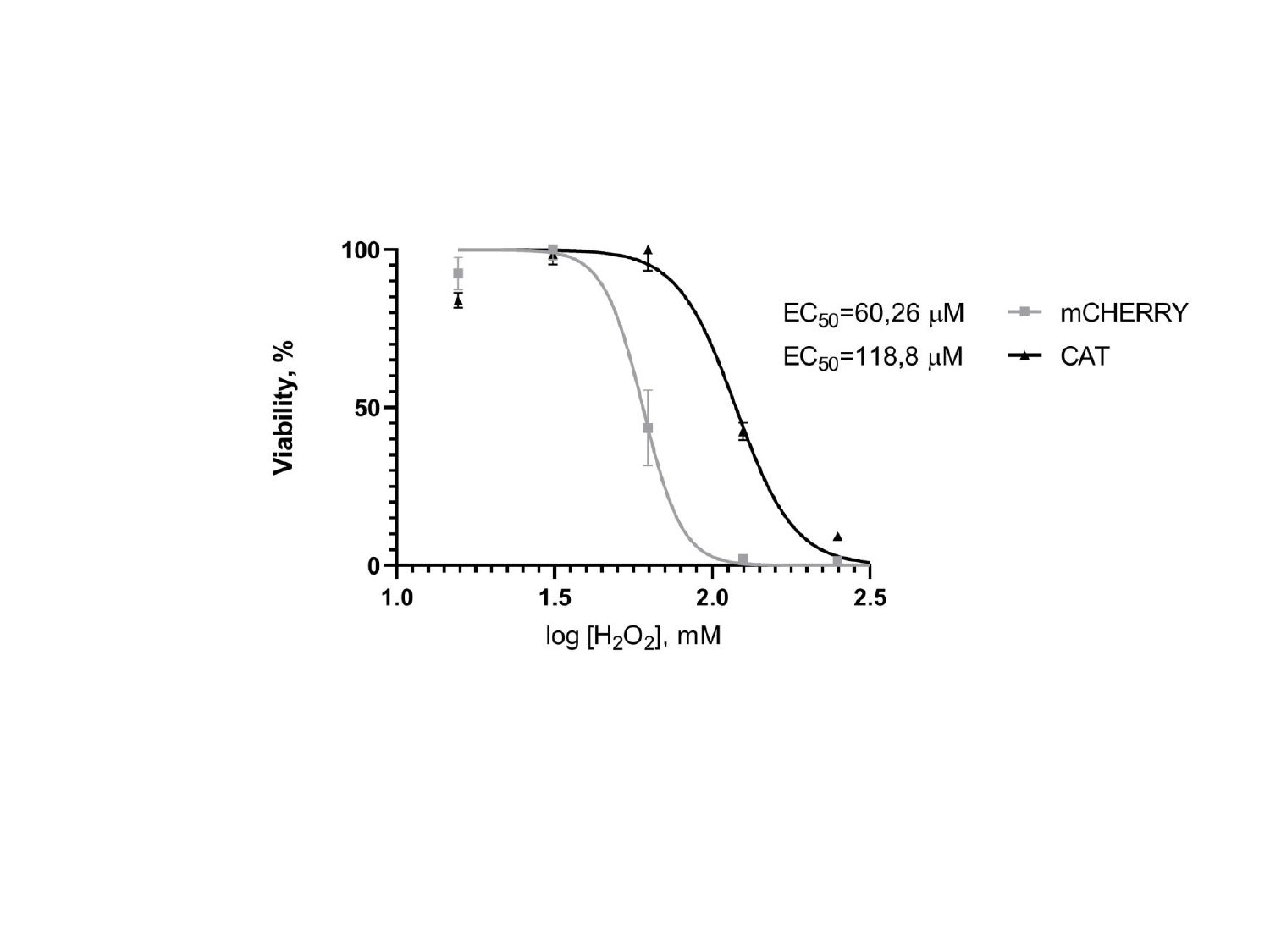

Supplement: Supplemental Material [file KVIR_A_1896830_SM4516.zip › S08 Fig R2.pptx]

## Slide 1
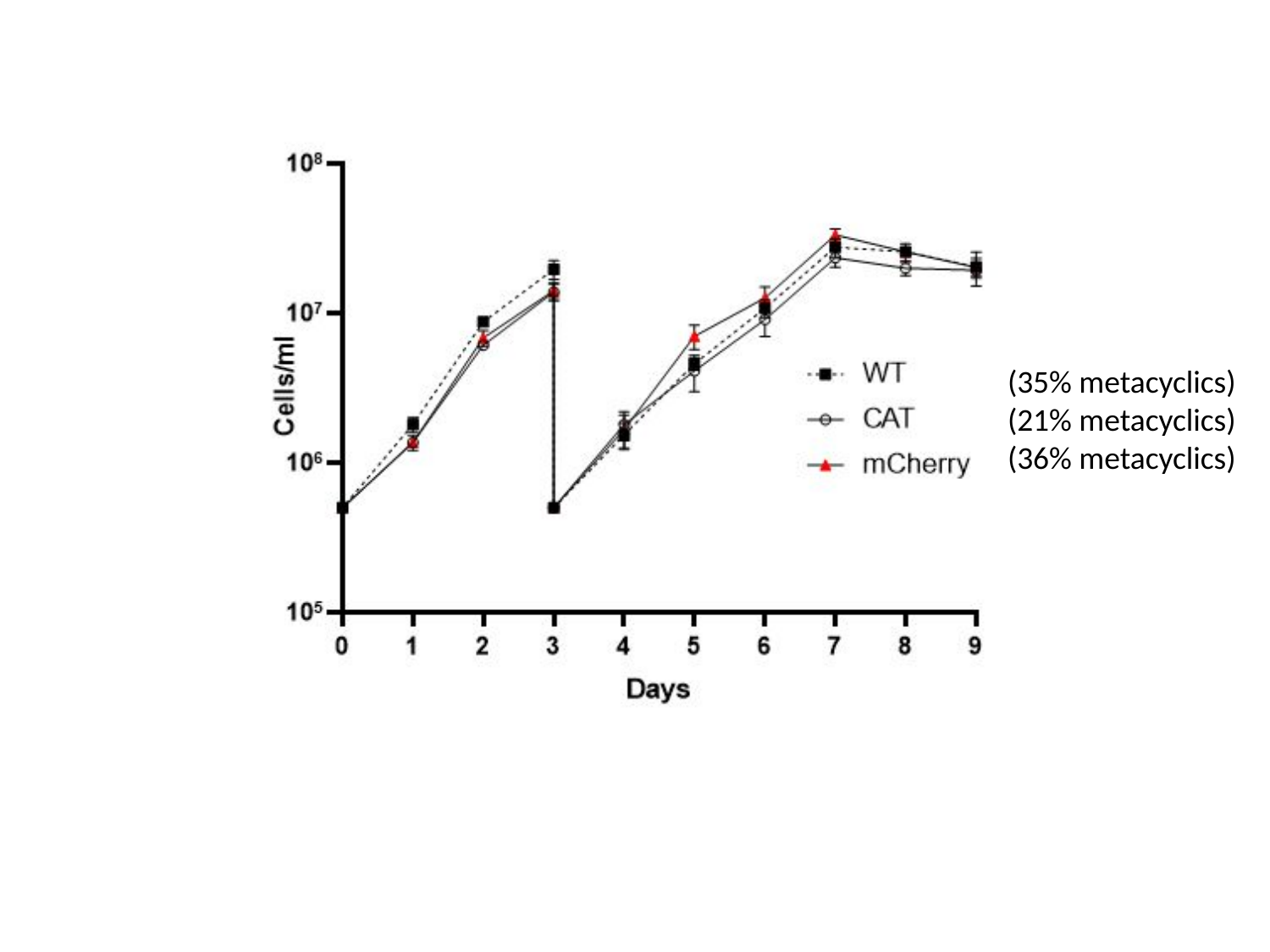

(35% metacyclics)
(21% metacyclics)
(36% metacyclics)

Supplement: Supplemental Material [file KVIR_A_1896830_SM4516.zip › S09 Fig R2.pptx]

## Slide 1
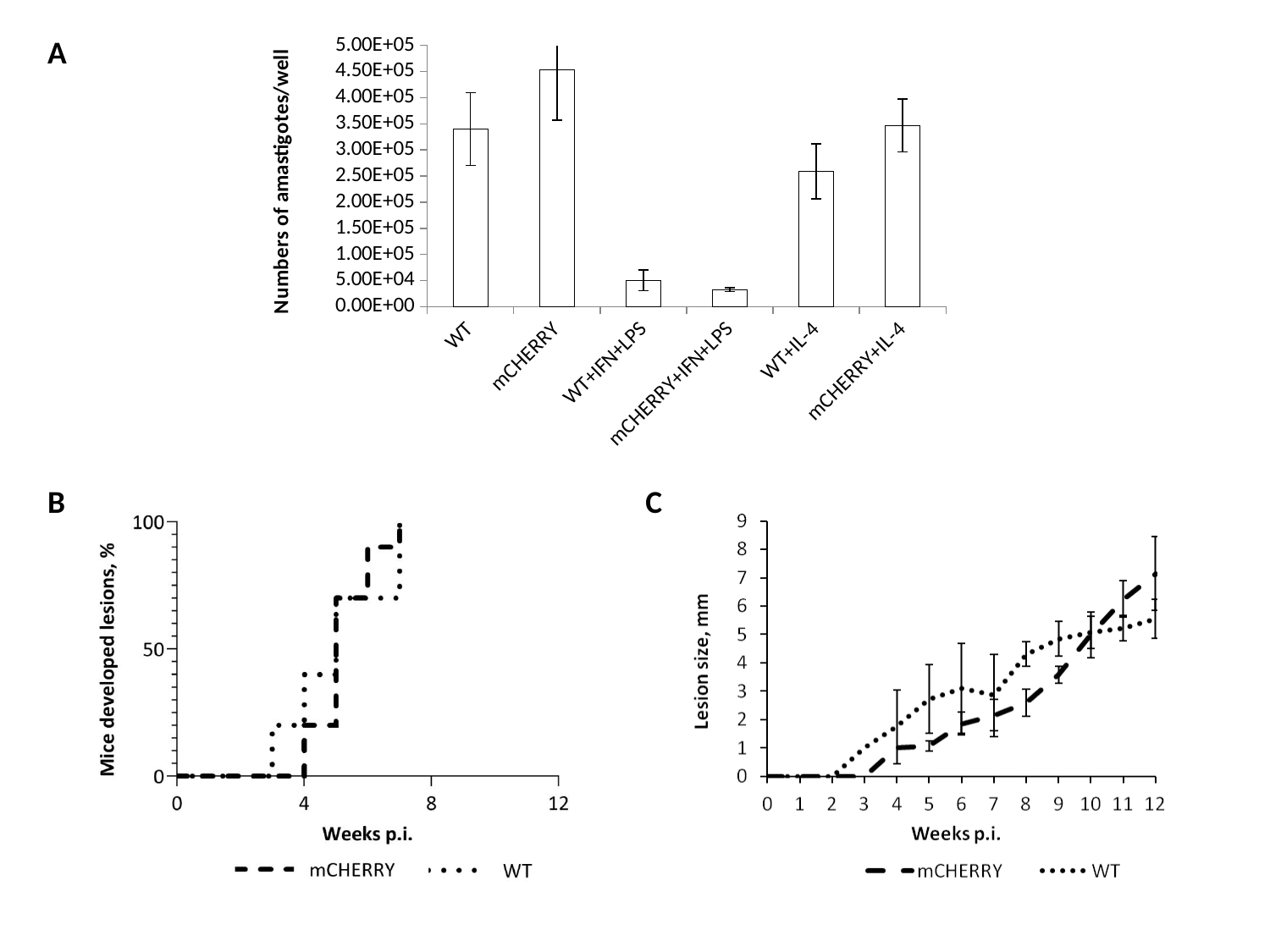

A
### Chart
| Category | |
|---|---|
| WT | 340000.0 |
| mCHERRY | 452500.0 |
| WT+IFN+LPS | 50333.333333333336 |
| mCHERRY+IFN+LPS | 32833.33333333333 |
| WT+IL-4 | 259166.6666666667 |
| mCHERRY+IL-4 | 346666.6666666666 |C
B

Supplement: Supplemental Material [file KVIR_A_1896830_SM4516.zip › S11 Fig R2.pptx]

## Slide 1
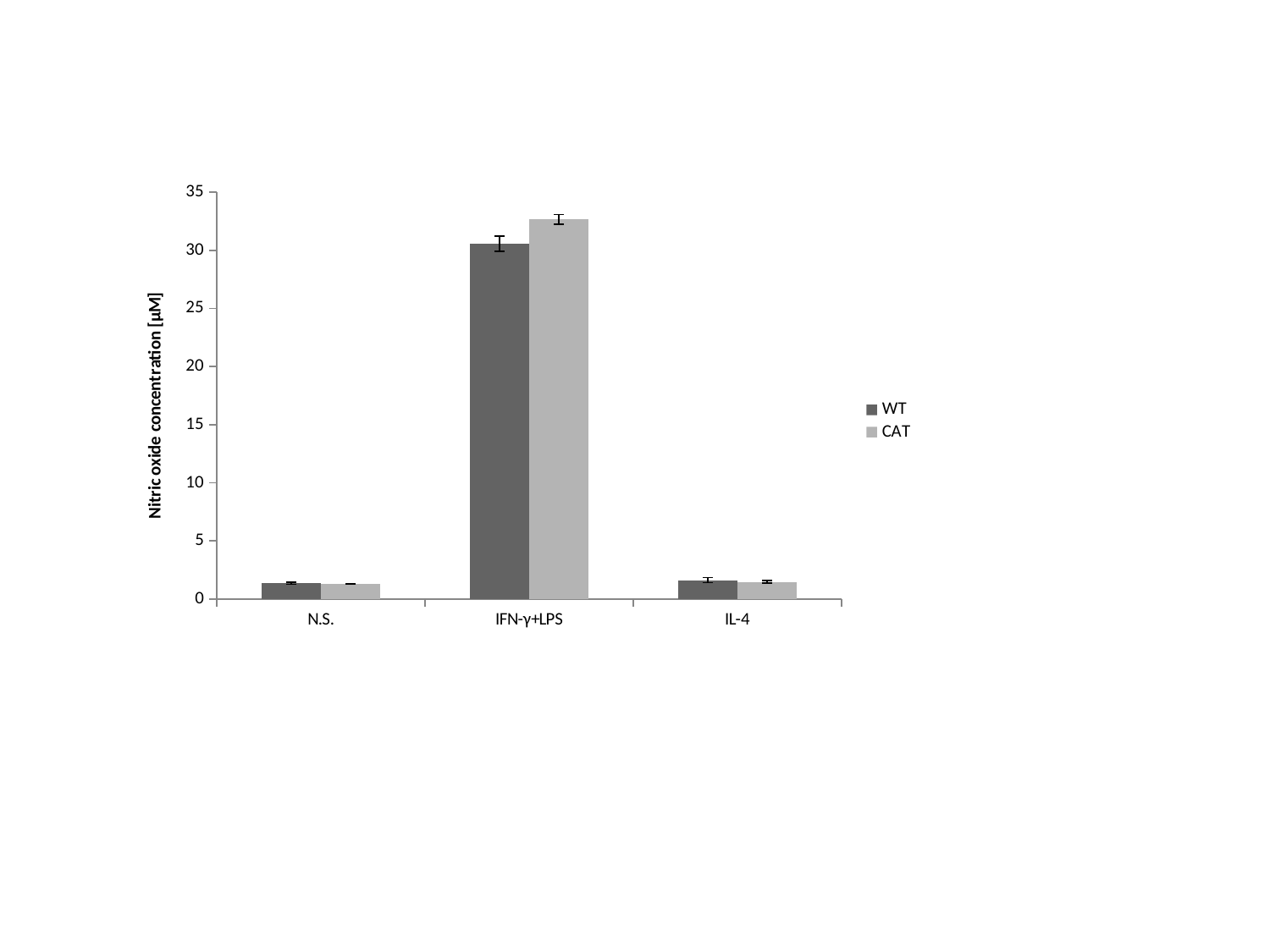

### Chart
| Category | WT | CAT |
|---|---|---|
| N.S. | 1.3612904294666663 | 1.2914895942666667 |
| IFN-γ+LPS | 30.57090796173333 | 32.659007325199994 |
| IL-4 | 1.633119723866667 | 1.484380756666667 |

Supplement: Supplemental Material [file KVIR_A_1896830_SM4516.zip › S12 Fig R2.pptx]

## Slide 1
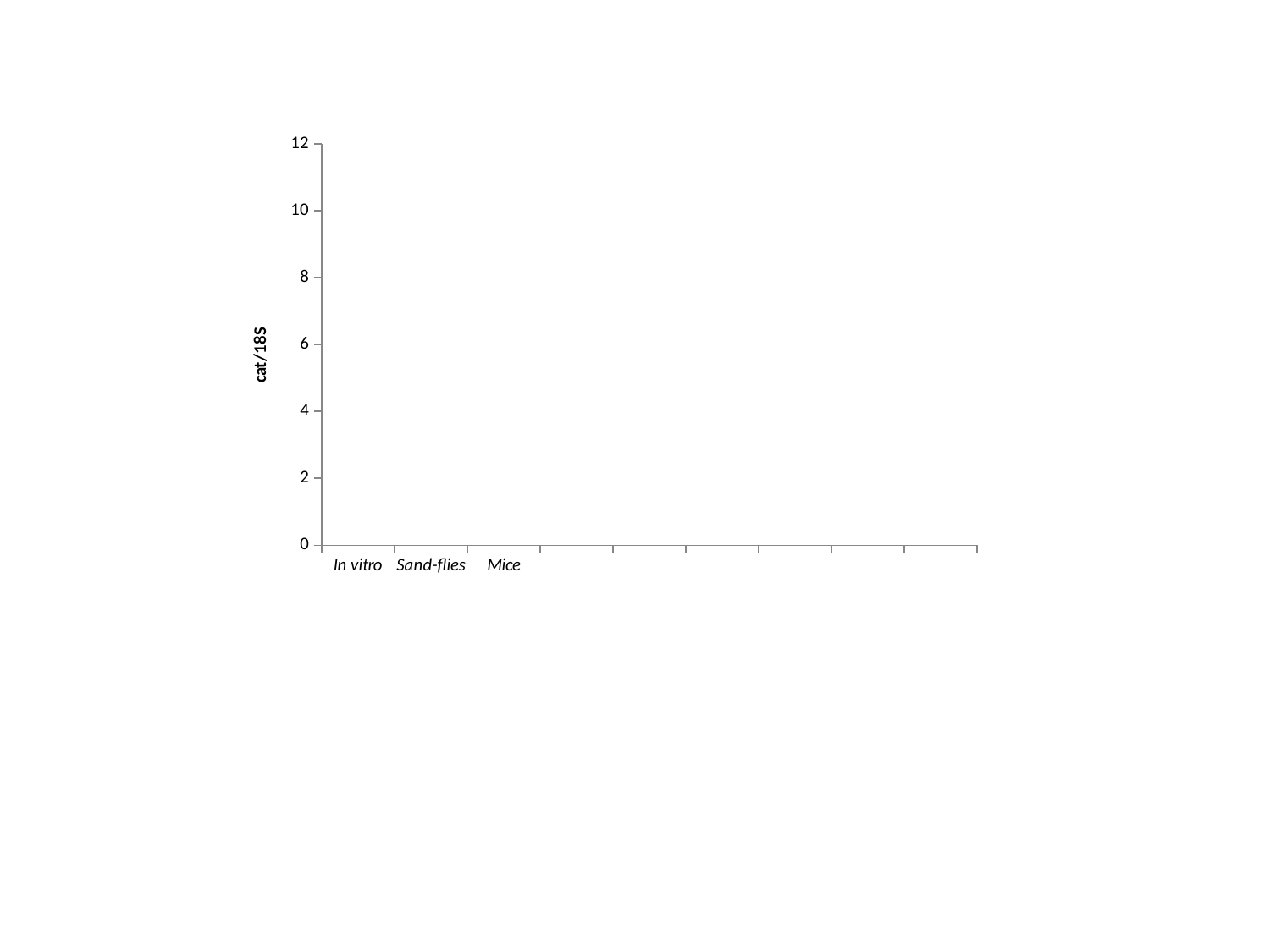

### Chart
| Category | |
|---|---|
| In vitro | 1.9027995005977212 |
| Sand-flies | 0.9541727419226204 |
| Mice | 0.9855175588622487 |

Supplement: Supplemental Material [file KVIR_A_1896830_SM4516.zip › S13 Fig R2.pptx]
